# Immune biomarkers predicting response to G-CSF in acute-on-chronic liver failure: results from a GRAFT trial sub-study

**DOI:** 10.1007/s12072-026-11069-5

**Published:** 2026-03-07

**Authors:** Katrin Splith, Nadja Berndt, Philipp K. Haber, Simon Wabitsch, Linda Feldbrügge, Adam Herber, Annegret Franke, Anett Schmiedeknecht, Jörg Mengwasser, Tony Bruns, Philipp A. Reuken, Tobias Goeser, Christoph Berg, Heiner Wedemeyer, Johannes Chang, Tobias Mueller, Niklas Aehling, Frank Lammert, Peter R. Galle, Z. Gordon Jiang, Simon C. Robson, Cornelius Engelmann, Thomas Berg, Moritz Schmelzle

**Affiliations:** 1 Department of General, Visceral and Transplant Surgery, Hannover Medical School, Carl-Neuberg-Str. 1, 30625 Hannover, Germany; 2 Department of Surgery, Campus Charité Mitte | Campus Virchow-Klinik, Charité-Universitätsmedizin Berlin, Berlin, Germany; 3 Division of Hepatology, Department of Medicine II, Leipzig University Medical Center, Leipzig, Germany; 4 Clinical Trial Centre (ZKS) Leipzig, Faculty of Medicine, University Leipzig, Leipzig, Germany; 5 Department of Medicine III, Aachen University Hospital, Aachen, Germany; 6 Clinic for Internal Medicine IV, University Hospital Jena, Jena, Germany; 7 Clinic for Gastroenterology and Hepatology, University Hospital Cologne, Cologne, Germany; 8 Department of Internal Medicine I, University Hospital Tübingen, Tübingen, Germany; 9 Department of Gastroenterology, Hepatology, Infectious Diseases and Endocrinology, Hannover Medical School, Hannover, Germany; 10 Department of Internal Medicine I, University Hospital Bonn, Bonn, Germany; 11 Department of Hepatology and Gastroenterology, Charité-Universitätsmedizin Berlin, Berlin, Germany; 12 Department of Medicine II, Saarland University Medical Center, Homburg, Germany; 13 Health Sciences, Hannover Medical School (MHH), Hannover, Germany; 14 Department of Internal Medicine, University Medical Center Mainz, Mainz, Germany; 15 Liver Clinic, Department of Medicine, Beth Israel Deaconess Medical Center, Boston, MA USA; 16 Department of Anesthesia, Critical Care and Pain Medicine, Center for Inflammation Research, Beth Israel Deaconess Medical Center, Boston, MA USA

**Keywords:** Liver failure, ACLF, Granulocyte-colony stimulating factor, Hematopoietic stem cells, Progenitor cells, Liver regeneration, Cytokines, Purinergic signaling, CD39, VEGF-A, Systemic inflammation

## Abstract

**Background:**

The efficacy of granulocyte-colony stimulating factor (G-CSF) treatment for acute-on-chronic liver failure (ACLF) remains controversial. The aim of this study, which is a secondary analysis of the GRAFT study (NCT02669680), was to identify potential prognostic biomarkers in ACLF and to find markers that could be used to predict response to G-CSF treatment.

**Methods:**

Blood samples from 79 patients randomized in the GRAFT study to receive G-CSF (*n* = 40) or standard medical therapy (*n* = 39) were collected at different timepoints. Samples were analyzed for cells, cytokines, extracellular particles, cell-free DNA, and functional properties. An exploratory approach was taken, whereby the measured variables were subjected to univariate and multivariate Cox analyses, and the patients were stratified into clusters by unsupervised hierarchical clustering.

**Results:**

Patients with ACLF had increased plasma levels of pro-inflammatory cytokines and increased absolute levels of specific cell populations when compared to healthy controls. ROC curve analysis suggested that plasma VEGF-A levels at baseline (timepoint) are a prognostic factor of transplant-free survival (AUC: 0.70; 95%CI 0.56–0.84). Hierarchical clustering of circulating immune cell populations at baseline appeared to define a patient subset within the analyzed G-CSF-treated patients with improved median transplant-free survival (102 days vs 16 days; *p* = 0.002). In our study, a higher percentage of CD39^+^ lymphocytes serves as an independent predictor of unfavorable outcomes following G-CSF treatment (HR: 1.05, 95%CI: 1.01–1.09, *p* = 0.008).

**Conclusion:**

VEGF-A appears to be a suitable biomarker for predicting the prognosis of patients with ACLF. With the help of hierarchical cell clusters, ACLF patients who could benefit from G-CSF therapy could be identified and selected prior to treatment. CD39 expression on lymphocytes seems suitable to allow stratification. Further testing and validation are necessary and could help to adapt treatment options for each individual patient.

**Graphical abstract:**

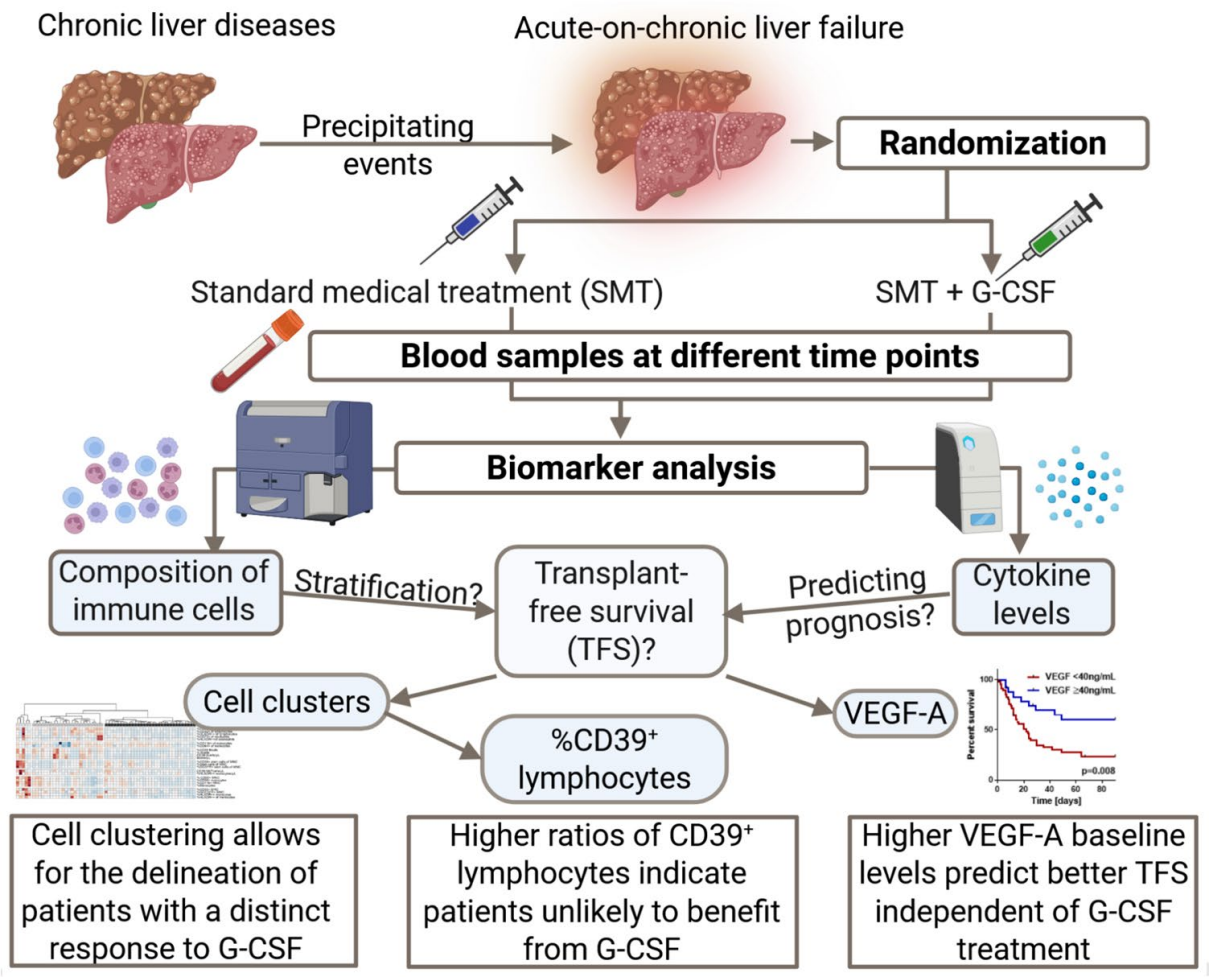

**Supplementary Information:**

The online version contains supplementary material available at 10.1007/s12072-026-11069-5.

## Introduction

Acute-on-chronic liver failure (ACLF) is defined by an acute insult in patients with cirrhosis. Acute deterioration of a preexisting chronic liver disease is typically related to a precipitating event, e.g., variceal bleeding, alcohol consumption, viral hepatitis or bacterial infections culminating in end-organ dysfunction [1–3]. Clinically, insults manifest as acute decompensation of cirrhosis complicated by additional organ failure [1, 2, 4]. Patients with underlying end-stage liver disease (ESLD) are characterized by immune dysfunction, which increases the susceptibility to infections [5]. This, in turn, aggravates pro-inflammatory responses that possibly lead to further deterioration of organ function. [6]

Granulocyte-colony stimulating factor (G-CSF) has been proposed as a potential candidate in the treatment of ACLF [7, 8]. Studies suggest that improved survival after G-CSF is associated with modulated innate immune functions and increased hepatic regenerative capacity. [7, 8] However, the efficacy of G-CSF in patients with ACLF remained controversial due to conflicting results from different studies in Asia and Europe [7–11]. Consequently, the GRAFT (granulocyte-colony stimulating factor to treat acute on-chronic liver failure: A multicenter randomized trial) study was conducted to investigate the efficacy and safety of G-CSF in an investigator-initiated, controlled trial, which recruited patients with ACLF at 18 German tertiary centers [9]. In contrast to previous studies, G-CSF had no beneficial effect, and the GRAFT study was discontinued after interim analysis due to futility. [9]

Nevertheless, the question of whether there are subpopulations of patients who might benefit from G-CSF remains open. It has been previously demonstrated that G-CSF mobilizes both immune and hematopoietic stem cells within the blood of patients presenting with liver failure [7, 12, 13] To address the importance of cellular responses to G-CSF and possible implications on ACLF, the GRAFT study was accompanied by a prospective biomarker study analyzing systemic cellular and molecular markers. The aim was to provide a comprehensive picture of hematopoietic stem and immune cell responses in patients with ACLF and to refine patient selection and monitoring procedures.

## Methods

### Study design

The GRAFT study (NCT02669680) was performed as a prospective, open-labeled, randomized, controlled, multicenter trial with 18 participating centers in Germany. The overall study design, protocol, and results of the study were published recently [9]. Between January 2016 and May 2019, the GRAFT study included 176 patients with age greater than 18 years and with ACLF, defined according to the EASL-CLIF criteria [14]. A full list of inclusion/exclusion criteria can be found in the online supplement.

**Fig. 1:**
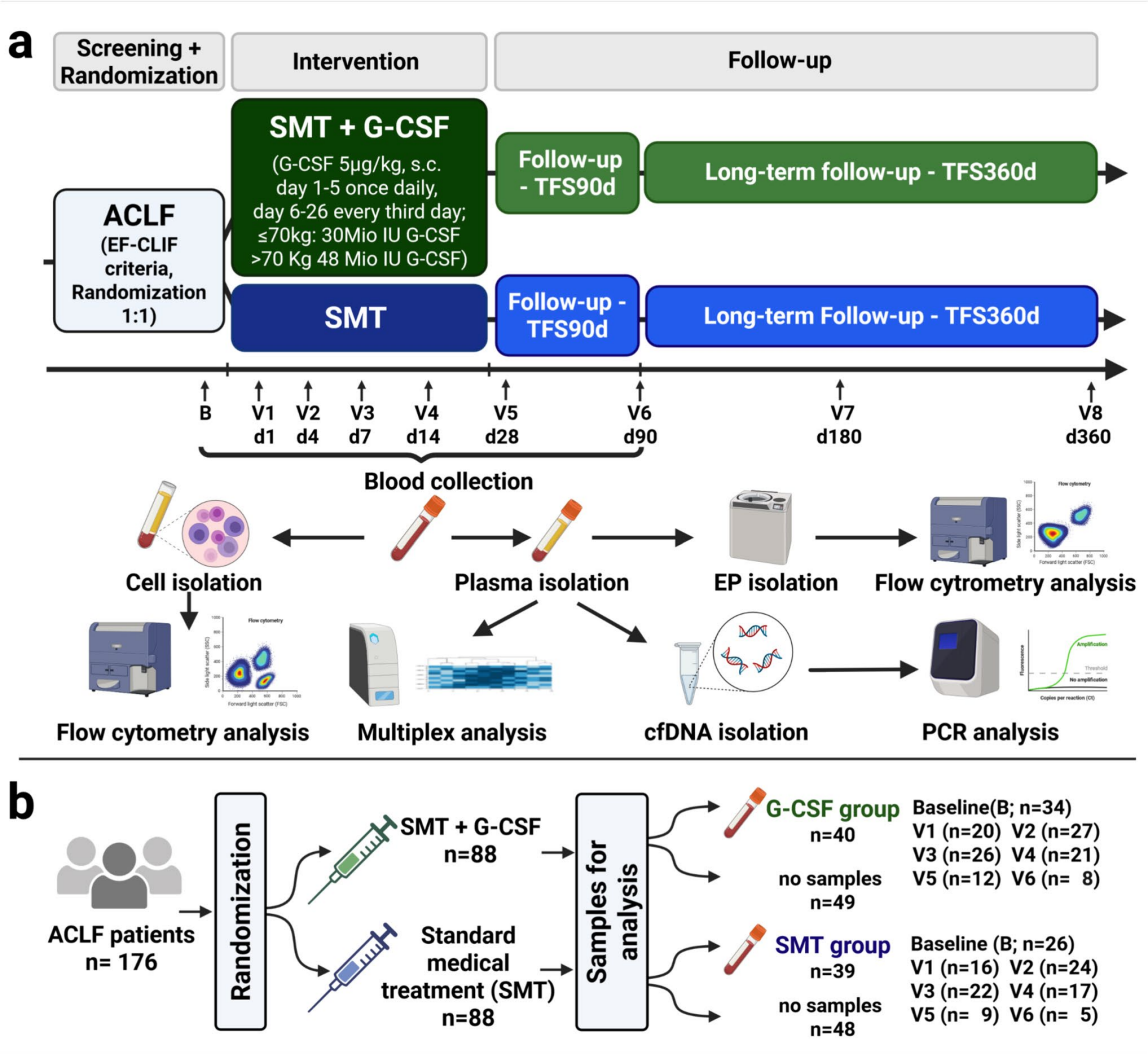
Study design and flow chart of the sub-study. **a** Schematic presentation of the ancillary study of GRAFT. ACLF patients were included in the GRAFT study and randomized into two groups, G-CSF and SMT [9]. Blood samples were collected at different timepoints (B-V6) and processed for analysis of blood cell populations, EP, cfDNA, and cytokines. **b** After the randomization of 176 patients (*n* = 88 patients in each arm), samples of 79 different patients at up to 7 different visits were included in this study. Samples of the other 97 patients were not available for our sub-study due to non-participation or shipping problems at some centers. At each timepoint, the stated number of samples could be analyzed; missing samples at any timepoint [B-baseline (before randomization); V1-day1; V2-day4; V3-day7; V4-day14; V5-day25; and V6-day90] are due to transportation issues or samples being unavailable

For the experimental intervention G-CSF (Filgrastim) in combination with standard medical therapy (SMT) of ACLF (G-CSF subcutaneously, 5 μg/kg daily day 0–4, then every 3rd day over 26 days (days 7, 10, 13, 16, 19, 22, 25) corresponds to 12 doses; experimental arm) was applied or SMT as control intervention (control arm) was implemented. Blood samples, and demographic and clinical data were collected (baseline (B; before treatment), after 1 day (V1), 4 days (V2), 7 days (V3), 14 days (V4), 28 days (V5), 90 days (V6), 180 days (V7), and 360 days (V8) or until liver transplantation/death) (Fig. 1a).

### Sample collection and analysis

Samples were shipped overnight on ice and were analyzed immediately via flow cytometry. Sample transport, preparation and analysis of cell populations, extracellular particles (EP), cell-free DNA (cfDNA), and cytokine levels as well as analysis of chemotactic activity, phagocytic activity, and oxidative burst of neutrophils and monocytes are described in the online supplement. Baseline values for cytokine expression, functional capacity, and a selected set of analyzed cell populations are displayed in Table S1.

### Statistical analyses

Statistical analyses were performed using SPSS statistics 23 (IBM, Chicago Illinois, USA), RStudio with R version 4.0.3 (Boston, MA, USA), and GraphPad Prism 6/8 (San Diego, USA). Data are presented as medians with interquartile ranges (IQRs). p values < 0.05 based on a two-tailed test were considered statistically significant (see online supplementary materials and methods).

## Results

### Baseline characteristics of the patients

Seventy-nine patients (45%) from the GRAFT study were included in this translational study (total of 176). Of the remaining 97 patients, no samples were received due to logistical reasons and could, therefore, not be included in this sub-study.

Forty of these 79 patients (51%) were treated with G-CSF in addition to standard care (G-CSF group), whereas 39 of 79 (49%) were treated with standard care only (SMT group; Fig. 1b). Demographic and clinical baseline characteristics are displayed in Table S2. There were no differences in baseline parameters or disease severity between patients included in our sub-study and those included in the overall GRAFT study. A table summarizing the baseline characteristics of these subgroups can be found in Table S3 of the supplemental material. Furthermore, no significant differences in routine clinical parameters were detected between the G-CSF group and the SMT group at baseline (Table S2).

Blood samples of ten healthy individuals with normal liver biochemistry tests and without evidence of hepatic or other diseases were included as controls.

### Identification of possible prognostic biomarkers in ACLF at baseline

Blood samples of 60 patients with ACLF were analyzed at baseline [G-CSF *n* = 34/40 (85.0%); SMT *n* = 26/39 (66.7%)]. Higher levels of pro-inflammatory cytokines were noted in ACLF patients when compared to healthy controls (Table S1). There were no significant differences in baseline parameters between the study groups (G-CSF vs. SMT; Table S1).

**Fig. 2:**
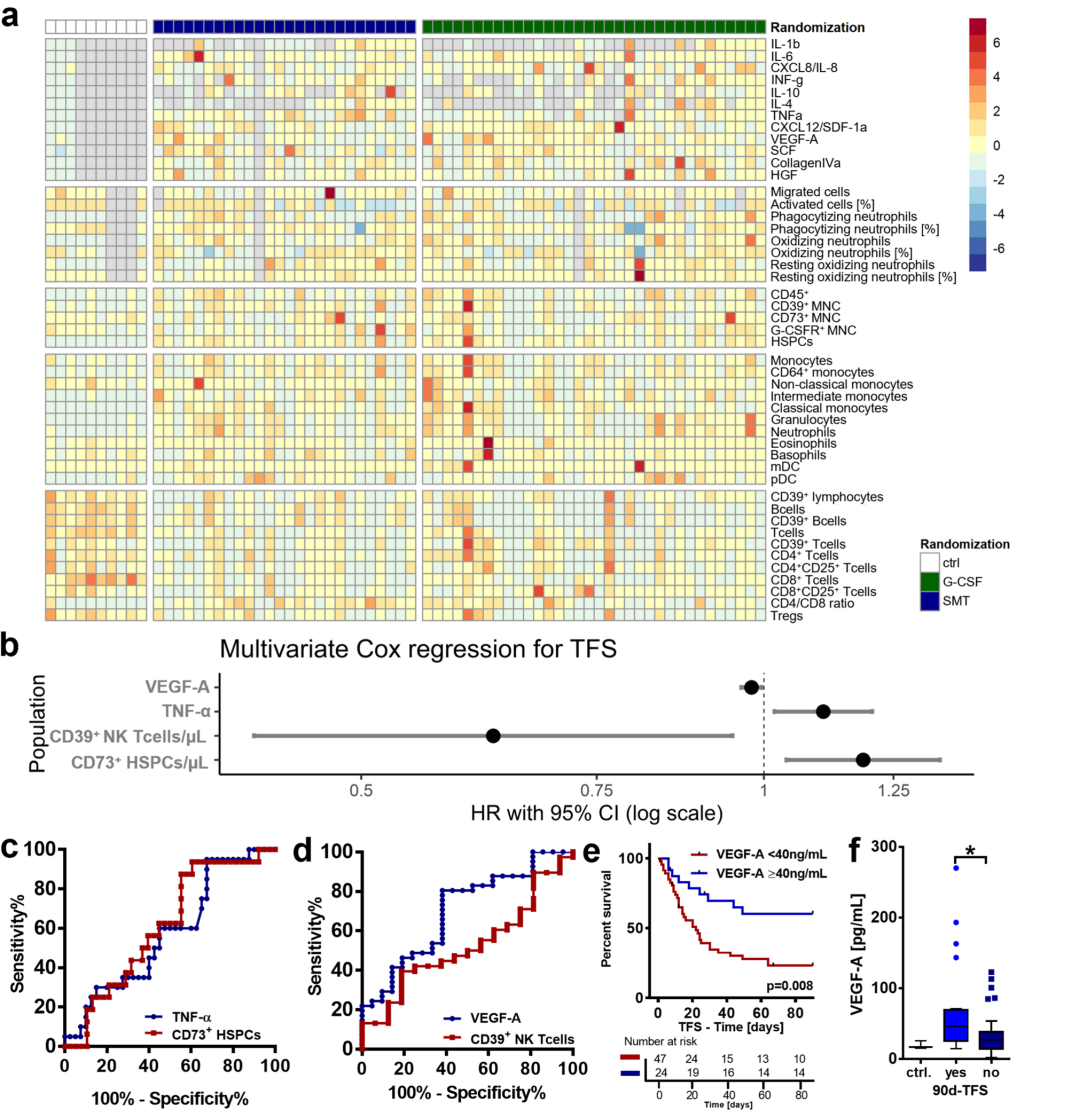
Baseline cell population and biomarker characteristics. **a** Heatmap of baseline characteristics of ACLF patients (G-CSF: *n* = 34; SMT: *n* = 26) and controls (*n* = 10). **b** Forest plot for multivariate Cox regression for VEGF-A, TNF-α, CD39^+^ NKT cells, and CD73^+^ HSPCs. **c** ROC curve for 90-day TFS (yes vs. no) by TNF-α (blue) and CD73^+^ HSPCs (red) at baseline. **d** ROC curve for 90-day TFS (yes vs. no) by VEGF-A (blue) and CD39^+^ NKT cells (red) at baseline. **e** Kaplan–Meier curves of VEGF-A level with optimal cutoff 40 ng/mL. **f** Baseline plasma VEGF-A levels of controls, 90-day survivors, and non-survivors

Absolute levels of T and B cells at baseline were found to be decreased (both *p* < 0.001) in ACLF patients, whereas overall absolute neutrophil (*p* < 0.001), monocyte (*p* < 0.001), and dendritic cell levels (*p* < 0.01) were increased when compared to healthy controls (Fig. 2a; Table S1). ACLF patients showed more neutrophils exhibiting oxidative burst (*p* = 0.001) and phagocytic activity (*p* = 0.007; Table S1); however, significant lower relative levels of cells were chemotactically activated when compared to control patients (*p* = 0.002; Table S1).

In univariate Cox analysis, 16 cell populations and two cytokines were found to be significantly associated with transplant-free survival (TFS) (Table S4). Subsequent multivariate Cox regression revealed that high baseline VEGF-A levels and high absolute levels of CD39^+^ NKT cells may indicate beneficial outcomes, whereas high baseline TNF-α levels and high absolute levels of CD73^+^ HSPCs may be associated with a poor prognosis (Fig. 2b). ROC curve analysis confirmed only plasma VEGF-A levels at baseline as an acceptable predictor of 90d-TFS in ACLF patients with an area under the curve (AUC) of 0.70 (95%CI 0.56–0.84) and a cut-off value of 40 ng/mL as defined by the Youden’s index. (Fig. 2c–f). Absolute CD39^+^ NKT cells (AUC: 0.53; 95%CI 0.36–0.69), absolute levels of CD73^+^ HSPCs (AUC: 0.58; 95%CI 0.43 to 0.74), and TNF-α levels (AUC: 0.61; 95%CI 0.46–0.77) were not found to be reliable predictors of TFS (Fig. 2c–d). Established ACLF prognostic markers demonstrate comparable prognostic values. In this cohort, the AUC for MELD and CLIF-C OF values was found to be 0.65 (95%CI 0.53–0.79) and 0.75 (95%CI 0.64–0.88), respectively. There was no prognostic value found for plasma levels of EPs and cfDNA.

### Cell population analysis at baseline can predict the benefit of G-CSF treatment

None of the routinely assessed clinical baseline parameters used in the GRAFT study (e.g., serum-bilirubin, serum-creatinine, MELD; CLIF-C OF) were able to predict a response to G-CSF in our cohort (Table S5). Therefore, we explored the potential of different cell populations, cytokines, EPs, and cfDNA.

**Fig. 3:**
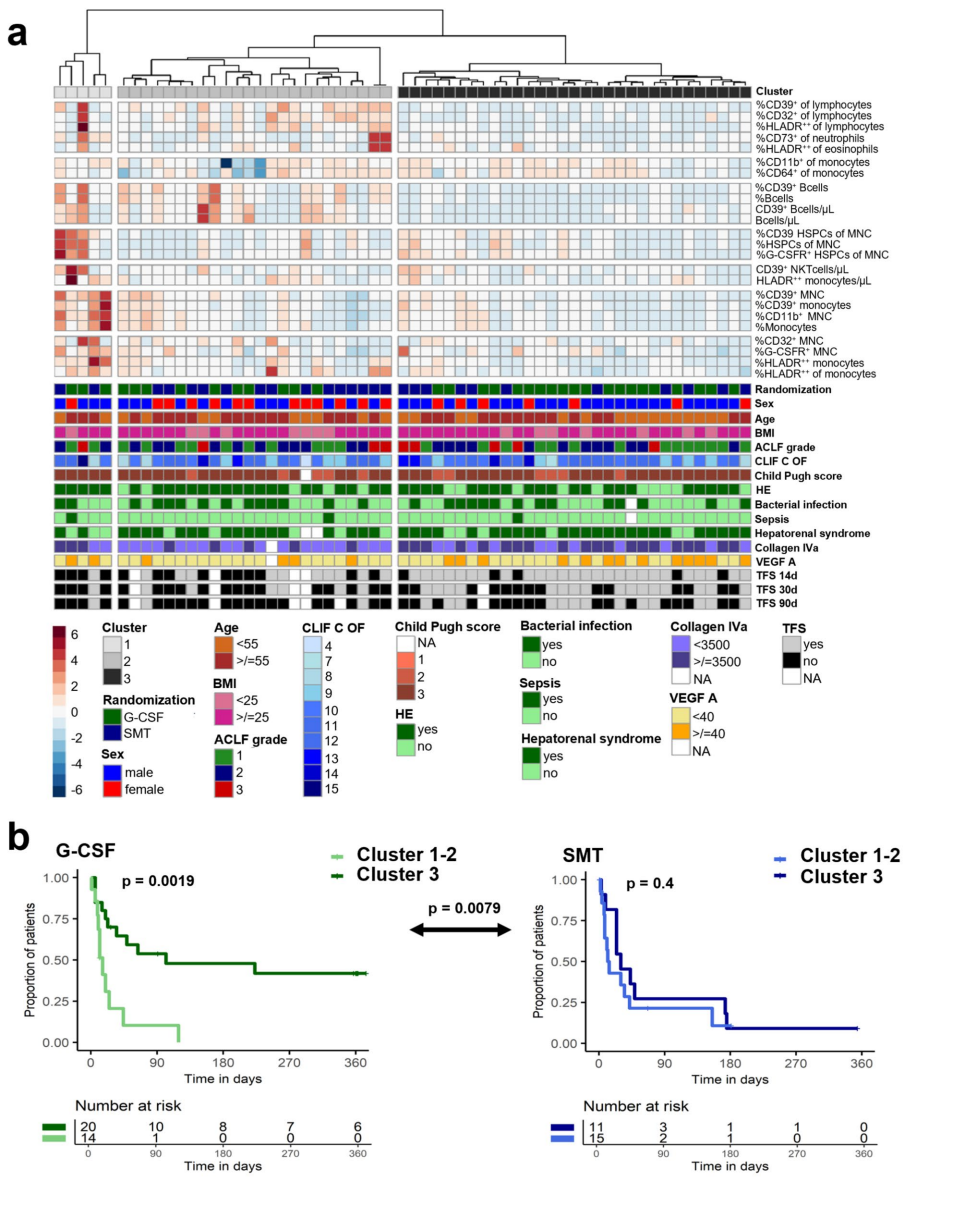
Unsupervised clustering based on cell populations at baseline. **a** Heat map of the prognostic baseline cell populations grouped by unsupervised clustering. ACLF patients (*n* = 60) with a similar baseline cell population pattern were clustered together [cluster 1 (*n* = 5), cluster 2 (*n* = 24), and cluster 3 (*n* = 31)]. Randomization, sex, age, BMI, ACLF grade, CLIF-C OF (CLIF-C organ failure score), Child–Pugh score, HE (hepatic encephalopathy), hepatorenal syndrome, bacterial infection, sepsis, collagen VIa, VEGF-A, and TFS for 14, 30, and 90 days are shown as annotations. **b** Survival curves for the two groups [clusters 1–2 (*n* = 29) and cluster 3 (*n* = 31)] of patients subdivided in the G-CSF and SMT groups; liver transplantation (OLT) was treated as a competing risk. P value of cell population treatment interaction was *p* = 0.0079. The statistical significances of the survival curves were analyzed using Kaplan–Meier method and log-rank test. Testing significance with Gehan–Breslow–Wilcoxon test results in similar significances (G-CSF: *p* = 0.013; SMT: *p* = 0.31; between groups: *p* = 0.04)

In univariate Cox analysis, 24 cell populations at baseline had a prognostic value for TFS in G-CSF patients. None of those cell populations had a prognostic value in SMT patients, suggesting that these were predictive of the G-CSF treatment and not merely a readout of prognosis in ACLF (Table S6). Euclidean clustering based on those baseline cell populations defined three subgroups (Fig. 3a): Patients in the first cluster (*n* = 5; 8.3%, cluster 1) show particularly vigorous immune responses to ACLF as a near global increase in baseline immune cells was observed.

Due to the limited sample size (*n* = 5) in cluster 1, this cluster was combined with cluster 2, given the observed similarities in cell populations and outcomes, for further analysis. Baseline characteristics for the clusters are displayed in detail in Table S7. Compared to cluster 3, pooled patients of clusters 1–2 show higher relative levels of CD39^+^ lymphocytes (*p* < 0.001), which is further reflected in higher absolute levels of CD39^+^ B cells (*p* = 0.0002) and CD39^+^ T cells (*p* = 0.01) as well as higher relative levels of CD39^+^ monocytes (*p* = 0.046). Higher relative levels of HLA-DR^+^ monocytes (*p* = 0.025) and lymphocytes (*p* = 0.001) are also characteristic of patients in clusters 1–2.

Overall, patients in cluster 3 treated with G-CSF had a median TFS of 102 days, whereas G-CSF-treated patients in clusters 1–2 had a median TFS of only 16 days (*p* = 0.002). No such discrepancies were observed in patients receiving SMT, with a median TFS in clusters 1–2 of 13 days vs. 24 days in cluster 3 (*p* = 0.4) (Fig. 3b). These data indicate that cluster 3 includes patients who are likely to benefit from G-CSF treatment [*n* = 31, (20 in G-CSF group, 11 in SMT group)], whereas those in clusters 1 and 2 are more likely to show no benefit [*n* = 29, (13 in G-CSF group, 16 in SMT group) or are even associated with worse outcomes after G-CSF treatment.

Plasma VEGF-A (*p* = 0.03), HGF (*p* = 0.02), and collagen IVa levels (*p* = 0.009) were significantly higher in cluster 3 when compared to patients in clusters 1–2 (Fig. 3b/Table S7). In contrast, no significant differences in standard clinical parameters, ACLF grade, or ongoing infections were observed between patients in clusters 1–2 and cluster 3 (Fig. 3b/Table S7).

### Differential response to G-CSF in ACLF patients

In addition to the baseline samples, we were able to analyze blood samples of 36 patients (*n* = 20 G-CSF group/*n* = 16 SMT group) at V1, 51 (27/24) at V2, 48 (26/22) at V3, 38 (21/17) at V4, 21 (12/9) at V5, and 13 (8/5) at V6. Patients show increased total leukocyte numbers after G-CSF treatment, demonstrated by a significant upregulation in most of the investigated cell populations (Table S8).

The cellular response to G-CSF treatment at V2 and V4 timepoints seems to be less pronounced in patients in clusters 1–2, which is particularly evident for absolute G-CSFR^+^ cells, HSPCs, and CD39^+^ lymphocyte levels (Supplementary Fig. 2a–d/Table S8).

Investigating plasma cytokine levels in G-CSF-treated patients, a significant decrease in CXCL8/IL-8 levels [*p* = 0.006 (B vs. V2)] and increase in HGF levels [*p* = 0.04 (B vs. V2)] over the time could be detected. Following G-CSF treatment, only patients in clusters 1–2 showed a significant increase in HGF (*p* = 0.02; Supplementary Fig. 2g), IL-6 (*p* = 0.02; Supplementary Fig. 2f), and TNF-α (*p* = 0.02; Supplementary Fig. 2h) levels, whereas only patients in cluster 3 showed a significant decrease in CXCL8/IL-8 (*p* = 0.03; Supplementary Figure 2e). In addition, G-CSF-treated patients showed an increase in plasma cfDNA levels [90 bp: 11.03 (4.12–35.90) ng/mL vs. 38.00 (14.20–70.97) ng/mL; B vs V2; Supplementary Fig. 2i], but values did not reach statistical significance.

Except for an increase in oxidizing and phagocytic neutrophils, which is in line with the overall increase in neutrophils after G-CSF treatment, no significant difference in the functional capacity of monocytes and neutrophils could be detected after G-CSF treatment and between the clusters (Table S8).

### CD39 expression on lymphocytes at baseline identifies patients who could benefit from G-CSF

A multivariate Cox analysis revealed that the relative levels of CD64^+^ monocytes and CD39^+^ lymphocytes at baseline were the only predictors for TFS depending on G-CSF treatment. While high relative levels of CD64^+^ monocytes seem to be associated with a clinical benefit (HR: 0.96, 95% CI: 0.93–0.99; *p* = 0.02) after G-CSF treatment, high relative levels of CD39^+^ lymphocytes could be associated with an adverse outcome after G-CSF treatment (HR: 1.05, 95% CI: 1.01–1.09, *p* = 0.008) (Table S4).

**Fig. 4:**
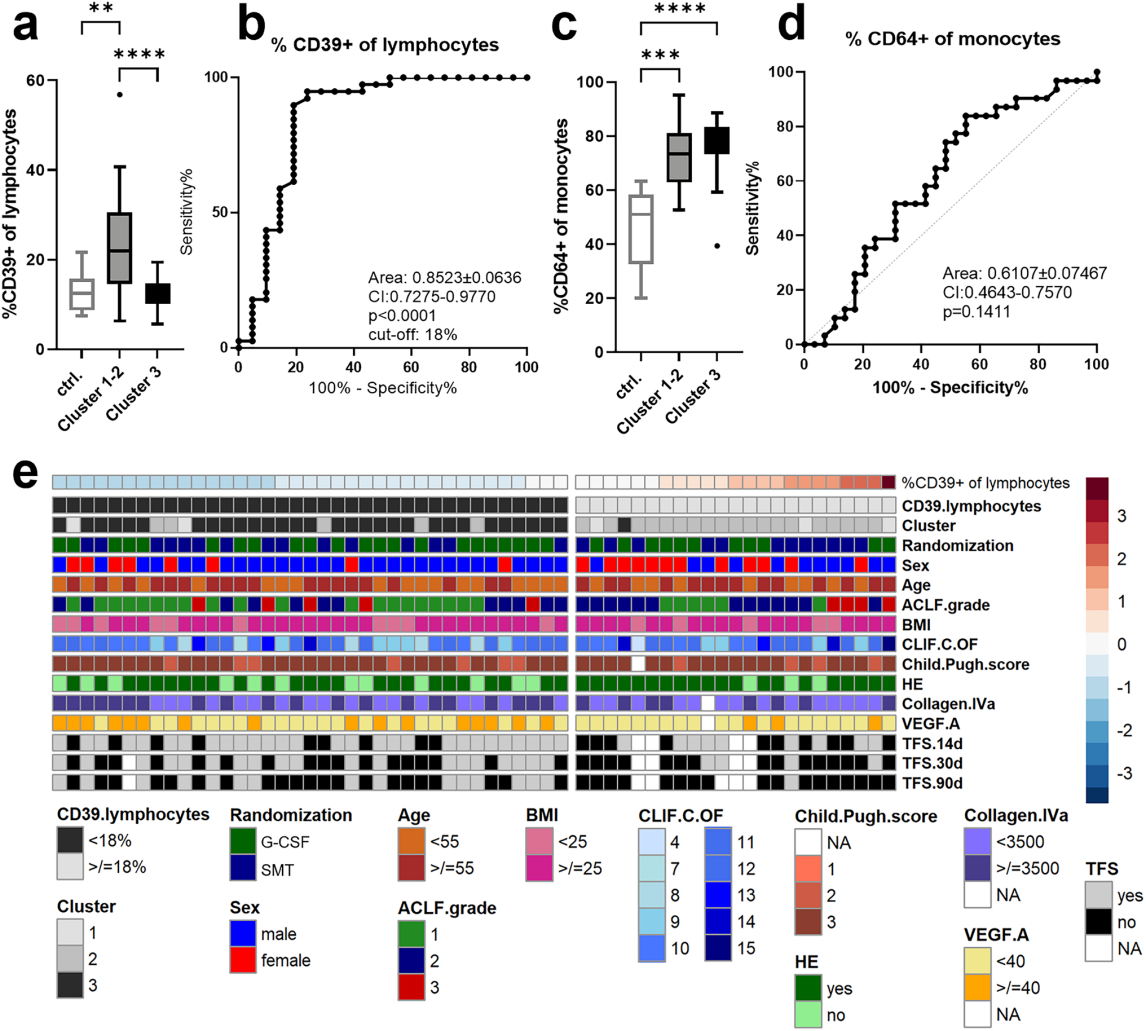
Classification of the patients by the relative levels of CD39^+^ lymphocytes or CD64^+^ monocytes **a** Relative levels of CD39^+^ lymphocytes in healthy controls (*n* = 10), patients in clusters 1–2 (*n* = 29), and cluster 3 (*n* = 31) at baseline. **b** ROC curve for classification into clusters (1–2 vs. 3) by relative levels of CD39^+^ lymphocytes with a cut-off value of 18% identified with the Youden’s index. **c** Relative levels of CD64^+^ monocytes in healthy controls (*n* = 10), patients in clusters 1–2 (*n* = 29), and cluster 3 (*n* = 31) at baseline. **d** ROC curve for classification into the clusters (1–2 vs. 3) by CD64^+^ monocytes. **e** Heatmap sorted by relative levels of CD39^+^ lymphocytes with a cut-off value of 18%. Randomization, sex, age, BMI, ACLF grade, CLIF-C OF, Child–Pugh score, hepatic encephalopathy (HE), collagen VIa, VEGF-A, and TFS for 14, 30, and 90 days are shown as patient annotations

We observed that patients in cluster 3 had a similar relative level of CD39^+^ lymphocytes at baseline compared to healthy patients, which served as controls, whereas those in clusters 1–2 displayed a markedly increased relative level of CD39^+^ lymphocytes at baseline compared to cluster 3 and healthy control patients (Fig. 4a, *p* < 0.0001, *p* = 0.004). ROC curve analysis for cluster classification of the patients showed that the relative level of CD39^+^ lymphocytes is a reliable predictor with an area under the curve (AUC) of 0.85 (95% CI 0.73–0.98) and a cut-off value of 18% identified with the Youden’s index (Fig. 4b).

Classification only based on relative levels of CD39^+^ lymphocytes almost corresponds to the classification based on the total cell population clusters (Fig. 4e) and shows a trend toward lower TFS for the G-CSF treated but non-benefitting patients (Supplementary Fig. 1a; *p* = 0.073), even at later timepoints of the G-CSF treatment (V2, *p* = 0.053) (Supplementary Fig. 1b). Conversely, classification based on CD64^+^ monocytes was unhelpful in predicting clinical outcomes after G-CSF treatment (AUC: 0.61, 95%CI 0.46–0.75, *p* = 0.141) (Fig. 4c–d, Supplementary Fig. 1c–d).

## Discussion

ACLF continues to be associated with a dismal outcome, and treatment focuses on attempts to eliminate the causative factors. Administration of G-CSF is known to promote hematopoietic stem and progenitor cell mobilization and has been investigated as a potential treatment option for ACLF.

Contrary to previous studies, the GRAFT study showed that G-CSF was futile in terms of prolonging both TFS and OS, and the study was stopped at the interim analysis [9]. The inter-trial heterogeneity in clinical outcome after G-CSF treatment, however, suggests that patient responses may be highly individualized. This prompted this exploratory molecular sub-study to characterize cellular and non-cellular responses in ACLF and to identify possible prognostic markers of disease progression and predictive markers in relation to G-CSF treatment.

Blood samples from ACLF patients were characterized by elevated inflammatory parameters, altered cell populations, and functional capacities compared to healthy controls. As a result of infections and elevated cytokine levels, ACLF patients displayed leukocytosis, with neutrophil (neutrophilia) and monocyte levels being particularly elevated [15]. In addition, a decrease in the frequencies of T cells, B cells, NK cells, and antigen-presenting cells (lymphopenia) can be seen during the development of ACLF [15, 16]. These cellular changes are thought to contribute to immune dysregulation and an overall immunosuppressive environment.

We found elevated baseline VEGF-A plasma levels in patients with ACLF compared to controls. Higher plasma VEGF-A levels were associated with beneficial outcomes in terms of 90-day TFS, comparable to established markers for ACLF outcome, such as MELD and CLIF-C OF. As the pro-angiogenic VEGF-A has previously been implicated in inducing vascular cell proliferation and migration as well as promoting leukocyte recruitment. These data suggest that functional VEGF-A regulation might be a driving factor in post-insult liver regeneration which may mitigate ACLF [17]. Standard cellular response patterns, EP and cfDNA levels, could not predict TFS or short-term survival in our cohort.

It is evident that patients suffering from ACLF frequently exhibit diminished chemotactic and phagocytic functionality in their neutrophils and monocytes [18]. Our findings demonstrate that these functions are downregulated in ACLF patients in comparison to healthy controls. It has been suggested that these functions can be enhanced through the administration of G-CSF [18]. However, our experiments showed no evidence of enhanced chemotactic, oxidative, or phagocytic function in these cells after G-CSF treatment despite an increased mobilization of neutrophils.

Given the variable results of clinical trials investigating the benefit of G-CSF in ACLF, [7–11, 19–22] we hypothesized that the reported heterogeneity may be reflected in individual patient outcomes, where G-CSF may be beneficial in some patients but not broadly applicable to all patients. Therefore, we investigated whether better clinical outcomes were associated with a reproducible molecular phenotype. Exploratory unsupervised clustering of the analyzed cell populations revealed two distinct subgroups, representing those patients who appear to benefit from G-CSF and, conversely, those who do not seem to benefit. Strikingly, patients within the two subgroups had similar outcomes when treated with SMT, underscoring the specific effect of G-CSF. In this scenario, it can be speculated that there may in fact be a subgroup of patients for whom treatment with G-CSF is detrimental, and that the varying presence of these patients in different cohorts may have contributed to the dissonance between different clinical trials. [7–11, 19–22]

Furthermore, our study demonstrated that those patients who exhibited a poor outcome after G-CSF administration also demonstrated decreased cellular responses in G-CSF. This observation suggests that the immune response in these patients may be compromised and they have a preexisting immune activation or even already approaching a state of immune exhaustion. This finding is consistent with reports from oncological settings, which indicate that G-CSF promotes an exhausted immune cell status and immune evasion [23]. Our data do not show a significant change in inflammatory markers in patients who seem to benefit from treatment with G-CSF. It may be assumed that the observed benefits are not attributable to the regression of inflammation or the altered function of granulocytes or monocytes. In patients who demonstrate a positive response, a substantial increase in overall mobilization of cells is observed. This mobilization includes HSPCs and neutrophils, which may result in an enhanced regenerative capacity [24, 25]. This mobilization is particularly effective in patients who do not yet show signs of preexisting immune activation or immune exhaustion.

Consequently, one may speculate that the use of G-CSF is unhelpful in patients who already have a preexisting immune activation. It is therefore critical to identify such patients prior to G-CSF administration. Conceptually, our data suggest that patients with ACLF can be stratified for different treatments at baseline using liquid biopsy analysis. However, as no differences in clinical characteristics were observed between the characterized patient subsets at baseline, the realization of precision medicine in this field will depend on the development of readily applicable biomarkers rather than the classification using complex measurements and cell clusters.

Using multivariate analysis, we found that CD39^+^ lymphocytes are able to distinguish subgroups of patients who will benefit from G-CSF treatment. These patients display rather low relative blood levels of CD39^+^ lymphocytes, similar to controls. CD39, also known as ectonucleoside triphosphate diphosphohydrolase 1 (E-NTPDase1), is expressed by activated and exhausted lymphocytes, dendritic cells, monocytes, and granulocytes. [26]

It has been shown that ongoing inflammation results in an increase in extracellular adenosine triphosphate (eATP), which is degraded by CD39 via CD73 to anti-inflammatory adenosine [17]. The release of eATP drives systemic inflammation [27], but is also a potent chemotactic and stimulatory factor for immune cells [28], which is necessary to control ongoing inflammation. High CD39 expression is associated with adenosine accumulation, immune cell exhaustion, and immunosuppression. [29, 30]

Due to the immunosuppressive effects of adenosine, patients with high levels of CD39^+^ lymphocytes may not be able to effectively fight ongoing infections, which are a major precipitating event in ACLF [4]. Additional administration of G-CSF could mobilize more suppressive effector cells in this group of patients, which may further amplify inflammatory events and lead to an unfavorable outcome. Maintaining the balance between eATP and adenosine appears to be critical to outcome. Therefore, the CD39-associated pathway might, therefore, be an interesting diagnostic and therapeutic target that requires further evaluation.

Recent studies have demonstrated that combinational therapies involving G-CSF, such as its use with TLR4 antagonist or prednisolone, might show enhanced efficacy in ACLF patients [31, 32]. Consequently, further studies are necessary to investigate the potential of modulating purinergic signaling pathways in combination with G-CSF.

Strengths and limitations: A major strength of the study is that it was conducted in a subset of patients who were randomized in the context of the phase II GRAFT trial [9]. GRAFT patients were followed prospectively, adverse events were managed, and outcomes and treatment were well studied. As in the general study, our subset is very heterogeneous in terms of preexisting conditions and events leading to ACLF, as well as medications. Unfortunately, as noted in the GRAFT paper, the etiology of the underlying chronic liver disease was not documented [9]. As we did not receive samples of all patients randomized in the GRAFT trial, we unfortunately have a small sample size within the groups which limits statistical power. There is also a risk of selection bias due to the fact that not all centers participated. To provide further validation of the biomarkers identified in this exploratory study and conduct more comprehensive analyses, it is essential to expand the cohort of patients with ACLF in a larger study.

## Conclusion

Plasma VEGF-A appears to be a suitable biomarker to predict prognosis in patients with ACLF and should be validated in larger cohorts. In our exploratory setting, we could identify patients with ACLF who may benefit from G-CSF prior to treatment using hierarchical cell clusters. Due to the impracticality of conducting complex cell analyses in standard clinical settings, an attempt was made to identify a biomarker capable of making this distinction. CD39 expression on lymphocytes seems to be a suitable candidate for this; however, its validity and reliability must be confirmed in a larger cohort. The ability to classify patients according to their likely response to G-CSF would help to better understand ACLF and adapt treatment options. Since personalized and combinational therapies will likely be needed to address the complexities of ACLF, a biomarker-enriched design for the next clinical trial might be suitable.

## Supplementary Information

Below is the link to the electronic supplementary material.

Supplementary file1 (DOCX 2226 KB)

## Data Availability

All data are available within the article, in supplementary information files, or from the author upon reasonable request.

## Abbreviations

ACLF: Acute-on-chronic liver failure
BfArM: German competent authority
CI: Confidence interval
cfDNA: Cell-free DNA
CLIF-C OF score: CLIF-C organ failure score
DC: Dendritic cell
DFG: Deutsche Forschungsgemeinschaft (German Research Foundation)
EASL-CLIF: European Foundation for the Study of Chronic Liver Failure
eATP: Extracellular adenosine triphosphate
EP: Extracellular particles
G-CSF: Granulocyte-colony stimulating factor
GRAFT: Granulocyte-colony stimulating factor to treat acute on-chronic liver failure: a multicenter randomized trial
HE: Hepatic encephalopathy
HSPCs: Hematopoietic stem and progenitor cells
HR: Hazard ratio
MELD score: Model of end-stage liver disease score
OLT: Orthotopic liver transplantation
SMT: Standard medical therapy
TFS: Transplant-free survival
WBC: White blood cell count
