# Supplementary material for: Immune biomarkers predicting response to G-CSF in acute-on-chronic liver failure: results from a GRAFT trial sub-study
Source: Hepatol Int. 2026 Mar 7;20(3):852–63. doi: 10.1007/s12072-026-11069-5 (PMC13332997; doi:10.1007/s12072-026-11069-5)
Supplement: Supplementary file 1 — Supplementary file1 (DOCX 2226 KB) [file 12072_2026_11069_MOESM1_ESM.pdf]

Supplementary Appendix

Immune biomarkers predicting response to G-CSF in acute-on-chronic liver failure – results from a GRAFT trial sub-study

Katrin Splith[1,2], Nadja Berndt[2], Philipp K. Haber[2], Simon Wabitsch[2], Linda Feldbrügge[1,2], Adam Herber[3], Annegret Franke[4], Anett Schmiedeknecht[4], Jörg Mengwasser[1,2], Tony Bruns[5,6], Philipp A. Reuken[6], Tobias Goeser[7], Christoph Berg[8], Heiner Wedemeyer[9], Johannes Chang[10], Tobias Mueller[11], Niklas Aehling[3], Frank Lammert[12,13], Peter R. Galle[14], Z. Gordon Jiang[15], Simon C. Robson[15,16], Cornelius Engelmann[3,11], Thomas Berg[3], Moritz Schmelzle[1,2]

Affiliations:

[1] Department of General, Visceral and Transplant Surgery, Hannover Medical School, Hannover, Germany

[2] Department of Surgery, Campus Charité Mitte | Campus Virchow-Klinik, Charité - Universitätsmedizin Berlin, Berlin, Germany.

[3] Division of Hepatology, Department of Medicine II, Leipzig University Medical Center, Leipzig, Germany.

[4] Clinical Trial Centre (ZKS) Leipzig, Faculty of Medicine, University Leipzig, Leipzig, Germany.

[5] Department of Medicine III, Aachen University Hospital, Aachen

[6] Clinic for Internal Medicine IV, University Hospital Jena, Jena, Germany.

[7] Clinic for Gastroenterology and Hepatology, University Hospital Cologne, Cologne, Germany.

[8] Department of Internal Medicine I, University Hospital Tübingen, Tübingen, Germany.

[9] Department of Gastroenterology, Hepatology, Infectious Diseases and

Endocrinology, Hannover Medical School, Hannover, Germany.

[10] Department of Internal Medicine I, University Hospital Bonn, Bonn, Germany.

[11] Department of Hepatology and Gastroenterology, Charité Universitätsmedizin Berlin, Berlin, Germany.

[12] Department of Medicine II, Saarland University Medical Center, Homburg, Germany;

[13] Health Sciences, Hannover Medical School (MHH), Hannover, Germany.

[14] Department of Internal Medicine, University Medical Center Mainz, Mainz, Germany.

[15] Liver Clinic, Department of Medicine, Beth Israel Deaconess Medical Center, Boston, Massachusetts, USA.

[16] Department of Anesthesia, Critical Care and Pain Medicine, Center for Inflammation Research, Beth Israel Deaconess Medical Center, Boston, Massachusetts, USA

Corresponding author:

Dr. Katrin Splith,

Hannover Medical School,

Department of General, Visceral and Transplant Surgery,

Carl-Neuberg-Str. 1, 30625 Hannover, Germany.

Table of content:

1. **Supplementary material and methods**
2. **Supplementary tables**
3. **Supplementary figure legends**
4. **Supplementary references**

1. Supplementary material and methods

In- and exclusion criteria (1)

Patients were included if the met all of the following criteria:

- Acute-on-chronic liver failure (ACLF) according to the consensus criteria recently defined by the CANONIC study group (2,3)
- Age ≥ 18 years, male or female
- Written informed consent from patient, legal or authorized representative or a confirmation of justification of trial participation by an independent medical consultant

Patients were excluded for if they met any of the following reasons:

- Prior not curatively treated or active malignancies
- Sickle cell disease
- Septic shock, defined by the following symptom complex: bacteraemia AND SIRS AND shock
- WBC-count of **>** 50 x 10^9/L
- Known HIV infection
- Known intolerance to filgrastim
- Suspected lack of compliance
- Pregnant or nursing women
- Fertile women (within two years of their last menstruation) without appropriate contraceptive measures (implanon, injections, oral contraceptives, intrauterine devices, partner with vasectomy) while participating in the trial (participants using a hormone-based method have to be informed of possible effects from the trial medication on contraception).
- Participation in other interventional trials

Blood samples

Blood samples (Citrate, EDTA and Heparin) from were collected at baseline (before randomization) to timepoint V6 at participating centers and sent to the laboratory in Berlin (Experimental Surgery, Charité – Universitätsmedizin Berlin, Berlin, Germany, Fig. 1b). Investigations regarding the stability of the samples under transport conditions were performed prior to the start of the study to verify the validity and reliability of the performed analyses.(data not shown) All cells were stained and analyzed by flow cytometry within 24h at the same day. The gating strategy and representative populations are displayed in Supplementary Fig. 3.

Citrate blood (baseline to V6) was centrifuged at 2500xg for 15 min and citrate plasma was immediately stored at -80°C. Furthermore, blood samples from 10 healthy controls were taken and processed in the same way. Heparin blood (B, V2, V4) was used for Phagotest®, Phagoburst® and Migratest®" assay according to the manufacturer's instructions (Glycotope Biotechnology GmbH, Heidelberg, Germany).

Cell isolation

Leukocytes from EDTA blood (baseline-V6) were obtained after lysis of the erythrocytes with ACK (ammonium-chloride-potassium) lysis buffer (150 mM NH_4Cl, 10 mM $NaHCO_3$, 0.1mM EDTA, pH 7.4) for 5 min at room temperature, followed by centrifugation. Cell pellets were washed once and resuspended in fluorescence-activated cell sorting (FACS) buffer (1% bovine serum albumin (BSA) and 0.1% NaN_3 in phosphate-buffered saline (PBS)).

cfDNA

The DNA extraction method employed here has been described previously by our group (4-5). Briefly, after thawing the samples at room temperature, plasma was centrifuged at 1000×g for 10min to remove any remaining components. For DNA isolation the QIAamp Blood DNA Mini Kit (Qiagen, Veno, The Netherlands) was used according to the manufacturer's protocol. After lysis of plasma samples, samples were washed several times. The elution buffer (50μL) was added, samples were incubated for 10min at room temperature. DNA was eluted from the column and stored at –20°C. Primer sets were selected to amplify two lengths of a multi-locus L1PA2 sequence. (6) The two DNA fragments, 90 and 222 bp long, were amplified (cf90 and cf222) with the same forward primer (5′-TGCCGCAATAAACATACGTG-3′) and different reverse primers (cf90: 5′-GACCCAGCCATCCCATTAC-3′ and cf222: 5′-AACAACAGGTGCTGGGAGAGG-3′).(6) Reactions contained 10μL GoTaq qPCR Master Mix (Promega, Fitchburg, MA, USA), 8μL nuclease-free water, 0.5μL forward primer (10μM) and 0.5μL reverse primer (10μM). One μL of cfDNA samples were added, and the thermal cycling conditions were as recommended by the manufacturer. PCR was performed as previously described on an Applied Biosystems 7500 real time PCR system. (4-5) Negative controls were included on each PCR plate to exclude possible contamination. A standard curve was prepared to determine the absolute concentration of cfDNA. Therefore, DNA was extracted from 200 μL whole blood as described above. Using the eluate, PCR was performed to obtain the corresponding fragment. We applied the Rapid PCR Cleanup Enzyme Set (New England Biolabs, Ipswich, MA, USA) to the amplified PCR products to degrade excess primers and dNTPs for purification of PCR products. The concentration of the DNA was measured using the Nanodrop 2000 (Thermo Fisher Scientific, Waltham, MA, USA) and a dilution series was placed in one row of the plate. The threshold cycle (Ct) value was determined for each dilution, and at least three dilutions (1:16000, 1:32000, 1:64000,

1:128000, 1:256000 or 1:512000) were measured for each reaction plate. The Ct value for each dilution was used to establish a standard curve for determining the cfDNA yield with each probe. The measurement was performed in duplicates.

Generation and isolation of extracellular particles

The generation and isolation of EP according to the protocol of Kornek et al. (7) was adopted previously by our group (8). Fluorescence-activated cell sorting (FACS) buffer (PBS, 1% BSA, 0.1% NaN_3) was filtered with a 0.2µm filter before use. Plasma samples were thawed, and 400µL of the plasma was transferred to a new 1.5 ml tube. The tube was filled with 1000µL filtered FACS buffer and centrifuged at 10000×g for 30min at 4°C to remove remaining platelets. Afterwards, 1300µL supernatant was slowly transferred to a microcentrifuge polyallomer tube with a snap-on cap (Beckman Coulter, Brea, CA, USA) and centrifuged at 100000×g for 95min at 4°C in an ultracentrifuge (Beckman Coulter, Optima XE Ultracentrifuge) to pellet EPs (7). The EP were resuspended in 400µL filtered FACS buffer, split into 100µL aliquots and stored at -80°C.

Flow cytomtery of cells

For flow cytomtery analysis 500000 cells were stained with antibodies in 3 different panels in combination with activation markers (9-10), ectonucleotidases (CD39/CD73)(11) and the G-CSF receptor (CD114)(12), respectively.

For the first panel (hematopoietic stem and progenitor cells(HSPCs)(13)), cells were stained with CD39-PECy7 (Biolegend, clone A1), CD41a-FITC (Biolegend, clone HIP-8), CD73-Pacific Blue (Biolegend, clone AD2), CD34-BV605 (Biolegend, clone 581), CD114(G-CSFR)-BV786 (Biolegend, LMM741), CD45-BV786 (Biolegend, clone HI30), and CD133-PE (Miltenyi, clone 293C3).

For the second panel(monocytes(14), DCs(15)), cells were stained with a lineage mix containing CD3-FITC (Biolegend; clone UCHT1) and CD19-FITC (Biolegend; clone HIB19), CD123-BV605 (Biolegend; clone 6H6), CD64-PE (Biolegend; clone 10.1), CD11b-PECy7 (Biolegend; clone ICRF44), CD32-APC (Biolegend, clone FUN-2), CD14-BV510 (Biolegend, clone M5E2), HLA-DR-BV711 (Biolegend, clone L243) and CD16-BV786 (Biolegend; clone 3G8).

For the third panel (B (16) and T (15) cell subsets), cells were stained with CD39-PECy7 (Biolegend, clone A1), CD16−BV786 (Biolegend; clone3G8), CD8a-FITC (Biolegend, clone RPA-T8), CD25-PE (Biolegend, clone BC-96), CD56-APC (Biolegend, clone HCD56), CD127-BV421 (Biolegend, clone A019D5), CD3-BV510 (Biolegend, clone UCHT1), CD19-BV605 (Biolegend, clone HIB19) and CD4-BV711 (Biolegend, clone Okt4).

All samples were stained for 20 min at 4° C in the dark, followed by centrifugation, washing and adding a viability dye (7-aminoactinomycin D (7-AAD), Biolegend) and counting beads (Biolegend, San Diego, CA, USA) adjusted according to manufacturer's instructions. Viable cells were gated based on forward scatter, sideward scatter and 7-AAD staining. The gating strategy and representative populations for each panel are displayed in Supplementary Fig. 4a-c.

Flow cytometry analysis of the blood cells was performed on a BD LSRFortessa™ X-20 (BD, Becton, Dickinson and Company, Heidelberg, Germany). Data were analyzed using FACSDiva™ (version 8.0.2) and FlowJo (version 10.4).

Flow cytometry of EP

Each sample containing 50µL supernatant EP and 5µL 0.2µm filtered 10x AnnV binding buffer (BD Biosciences, Heidelberg, Germany, Cat. No. 556454) was subsequently incubated with antibodies: APC Alexa 700-conjugated Cx43 (clone:

FAB7737N, R&D Systems, Minneapolis, MN, USA), BV421-conjugated CD130 (clone: AM64, BD Biosciences, San Jose, CA, USA), and PE-conjugated AnnV (Cat. No. 640908, BioLegend, San Diego, CA, USA), and FITC-conjugated ASGR1 (clone: REA608, Miltenyi Biotec, Auburn, CA, USA) or FITC-conjugated CD31 (Cat. No. 303104, BioLegend). The samples were incubated for 20 min at 4° C in the dark and then preserved on ice until measurement.

The characterization and counting of EP were achieved by flow cytometry as investigated by Kornek et al. (7) and described by our group.(8) Prior to measurement, the flow cytometer was rinsed with FACS buffer that had been prefiltered through a 0.2µm filter (Sartorius, Göttingen, Germany, Cat. No. ST16534-K). Filtered FACS buffer and only-antibody samples with FACS buffer were recorded to identify remaining background events. Each EP sample was mixed evenly with 25µL counting beads (Biolegend, San Diego, USA, Cat. No. 424902). The gating strategy and representative populations are displayed in Supplementary Fig. 4d.

Flow cytometry analysis was performed on a BD LSRFortessa™ X-20 (BD, Becton, Dickinson and Company, Heidelberg, Germany). Data were analyzed using FACSDiva™ (version 8.0.2) and FlowJo (version 10.4).

Phagotest®, Phagoburst® and Migratest®

The Phagotest®, Phagoburst® and Migratest® kit (Glycotope Biotechnology GmbH, Heidelberg, Germany) were used according to manufacturer's instructions to determine the amount and percentage of neutrophils and monocytes that phagocytize, produce reactive oxidants, and the amount and percentage of cells that migrate with and without stimulation. Flow cytometry analysis of the assays was performed on a BD

LSRFortessa™ X-20 (BD, Becton, Dickinson and Company, Heidelberg, Germany). Data were analyzed using FACSDiva™ (version 8.0.2).

Plasma biomarker multiplex assay

Plasma concentrations of CXCL12/stromal cell-derived factor-α (SDF-1a), hepatocyte growth factor (HGF), stem cell factor (SCF), vascular endothelial growth factor-A (VEGF-A), collagen IVa, interferon-γ (IFN-g), interleukin (IL)-1b, IL-4, IL-6, CXCL8/IL-8, IL-10, and tumor necrosis factor-α (TNF-a) were measured in citrate plasma in duplicate using the MagPix® System, a multiplex platform (Luminex Corporation, Madison, WI, USA) with a customized cytokine kit (R&D Systems, Inc, Minneapolis, MN, USA) and processed according to standard protocol. The 12 plasma biomarkers were chosen a priori as possible liver failure dependent indicators.

Statistical analysis

Depending on whether they are normally distributed or not normally distributed, two groups were compared using the t-test, the Mann-Whitney U test or Wilcoxon matched-pairs signed rank test. If more than 2 groups were compared, we used the 1-way analysis of variance (ANOVA), Kruskal-Wallis tests or Friedman tests as appropriate. For categorical variables, comparisons between groups were made using the Chi-squared tests, or the Fischer test. The Kaplan-Meier method was used to calculate the survival probability curves, which were compared with a log-rank test. Cox regression models were used for uni- and multivariate analysis of outcome predictors. For the screening of all cell populations, we calculated the multiple comparison-adjusted p-value using the False Discovery Rate (FDR) with Benjamini-Hochberg (BH) method, to control the increased chance of false positives (Type I errors). Prognostic value and

predictors were confirmed and cut-off values were determined using receiver operating characteristics (ROC) analysis and the Youden’s index. The heatmap for of the cell populations was generated by R package of pheatmap. Furthermore unsupervised hierarchical clustering analysis was performed using the pheatmap package with a “euclidean” distance and a “ward.D” linkage option to assess similarity and construct the clustering dendrogram.

2. Supplementary tables

Table S1: Biomarker baseline characteristics

Parameter						
Cytokines	**Ctrl. (n=3)**	**All patients (n=60)**	**p**	**G-CSF (n=34)**	**SMT (n= 26)**	**p**
IL-1b [pg/mL]*	**0.58 (0.38-0.68)**	**2.84 (1.18-3.97)**	**0.049**	3.3 (1.5-5.4)	2.1 (0.8-3.8)	n.s.
IL-6 [pg/mL]*	**1.02 (0.87-1.15)**	**20.2 (10.4-35.64)**	**<0.001**	19.6 (10.0-26.8)	24.5 (15.0-36.9)	n.s.
CXCL8/IL-8 [pg/mL]*	**14.6 (8.7-20.6)**	**41.5 (18.8-88.4)**	**0.03**	40.1 (14.2-113.3)	42.8 (21.0-76.2)	n.s.
IFN-g [pg/mL]*	<3.55	8.9 (5.2-16.5)	-	10.4 (7.1-1 5.0)	8.5 (4.4-16.7)	n.s.
IL-10 [pg/mL]*	<0.25	1.7 (0.97-2.9)	-	1.4 (1.0-2.5)	2.1 (1.2-3.8)	n.s.
IL-4 [pg/mL]*	<0.25	27.1 (12.4-53.8)	-	46.1 (20.6- 74.1)	26.2 (8.8-31.8)	n.s.
TNF-a [pg/mL]*	**0.40 (0.20- 0.59)**	**6.1(3.4-8.3)**	**<0.001**	6.0 (3.3-7.7)	6.3 (3.4-8.9)	n.s.
CXCL12/SDF-1a [pg/mL]*	**224.0(192.4-282.6)**	**941.4(510.0-1312.9)**	**0.006**	943.3(615.9-1250.0)	848.4 (427.0-1332.3)	n.s.
VEGF-A [pg/mL]*	19.5 (16.3-21.4)	29.29 (19.0–51.14)	n.s.	33.0 (23.3-59.7)	26.0 (16.2-39.2)	n.s.
SCF [pg/mL]*	**47.3 (42.2-56.7)**	**138.1 (96.5-186.7)**	**0.001**	138.7 (104.9-172.0)	135.2 (96.1-190.1)	n.s.
Collagen IVa [pg/mL]*	**536.5(307.4-691.8)**	**3326.8 (1959.1 -8428.2)**	**<0.001**	4093.3(2016.8-8911.3)	2304.6(1775.3-5899.0)	n.s.
HGF [pg/mL]*	**152.0(108.5-218.3)**	**2013.3(848.0-4090.2)**	**<0.001**	2241.8(868.2-5213.9)	1965.9 (826.9-3190.6)	n.s.
Functional capacity	**Ctrl. (n=7)**	**All patients (n=60)**	**p**	**G-CSF (n=34)**	**SMT (n= 26)**	**p**
Phagocytizing neutrophils [x10^6/L]*	**1912.1 (1565.6-2379.5)**	**4095.0 (2342.7-7704.9)**	**0.007**	3747.8 (2279.8-8726.8)	4504.6 (2325.4-7422.7)	n.s.
Phagocytizing neutrophils [%]*	**96.3 (95.9-97.9)**	**93.0 (86.7-96.1)**	**0.03**	93.5 (86.3-95.1)	91.7 (86.9-96.7)	n.s.
Resting oxidizing neutrophils [x10^6/L]*	**7.05 (4.05-12.19)**	**45.08 (19.05-95.83)**	**<0.001**	52.1 (28.3- 92.6)	41.1 (11.4-100.8)	n.s.
Resting oxidizing neutrophils [%]*	0.3 (0.2-0.6)	1.1 (0.3-2.0)	n.s.	1.25 (0.4-2.0)	1.1 (0.2-1.8)	n.s.
Oxidizing neutrophils [x10^6/L]*	**1628.9 (1122.8-2022.2)**	**3673.2 (2419.7-6544.6)**	**0.001**	3536.6 (2369.6-6964.0)	3673.1 (2443.5-6109.8)	n.s.
Oxidizing neutrophils [%]*	87.2 (73.8-94.2)	88.5 (72.3-93.7)	n.s.	89.5 (78.0-93.8)	86.3 (64.9-93.6)	n.s.
Ratio migrated cells [test/ctrl]*	2.12 (1.54-3.38)	1.42 (0.43-2.77)	n.s.	1.24 (0.53-2.01)	2.38 (0.40-3.60)	n.s.
Chemotactically activated cells [%]*	**99.7 (99.65-99.8)**	**89.2 (15.0-99.2)**	**0.002**	91.5 (22.6-99.3)	79.6 (2.7-99.0)	n.s.
Phagocytizing monocytes [x10^6/L]*	255.4 (102.4-284.1)	274.1 (162.4-511.2)	n.s.	299.0 (167.7-530.6)	273.6 (175.2-443.8)	n.s.
Phagocytizing monocytes [%]*	86.2 (73.1-91.9)	65.9 (56.8-81.8)	n.s.	73.1 (51.6-81.6)	74.7 (64.5-82.2)	n.s
Resting oxidizing monocytes [x10^6/L]*	1.4 (0.9-1.9)	4.6 (1.2-11.2)	n.s.	4.2 (1.2-10.4)	4.5 (1.2-13.9)	n.s.
Resting oxidizing monocytes [%]*	0.6 (0.4-0.8)	1.2 (0.2-3.0)	n.s.	1.2 (0.2-3.1)	1.1 (0.2-2.8)	n.s.
Oxidizing monocytes [x10^6/L]*	27.8 (16.6-41.6)	132.3 (41.4-234.9)	n.s.	143.4 (63.2-229.3)	116.6 (30.2-301.8)	n.s.
Oxidizing monocytes [%]*	7.1 (4.7-21.9)	39.6 (11.3-55.3)	n.s.	41.2 (15.6-52.5)	35.8 (10.7-63.4)	n.s.
Cell populations	**Ctrl. (n=10)**	**All patients (n=60)**	**p**	**G-CSF (n=34)**	**SMT (n= 26)**	**p**
CD45+ [x10^6/L]*	**4128.2 (2815.7-4479.0)**	**5881.1 (3633.5-10618.2)**	**0.02**	6226.9 (3377.6-10360.6)	5646.7 (3756.6-11596.0)	n.s.

CD73+ MNC [x10^6/L]*	90.7 (78.4-130.4)	85.6 (44.8-155.0)	n.s.	85.0 (52.2-161.0)	85.6 (34.4-104.6)	n.s.
CD73+ of MNC [%]*	8.9 (6.0-9.9)	8.0 (4.8-13.6)	n.s.	7.8 (5.2-11.9)	8.4 (3.6-15.0)	n.s.
CD39+MNC [x10^6/L]*	**244.4 (190.9-317.2)**	**486.6 (267.1-588.0)**	**0.01**	503.4 (263.8-577.4)	439.8 (290.0-714.2)	n.s.
CD39+ of MNC [%]*	**22.2 (17.7-25.7)**	**49,2 (40.9-56.0)**	**<0.0001**	49.2 (39.7-52.6)	49.7 (42.2-58.7)	n.s
G-CSFR+ MNC [x10^6/L]*	231.1 (172.8-252.6)	216.4 (150.5-389.2)	n.s.	216.4 (149.2-402.2)	231.9 (153.7-378.8)	n.s.
G-CSF+ of MNC [%]*	**14.0 (12.9-25.0)**	**25.7 (19.7-34.9)**	**0.001**	25.6 (19.0-36.4)	25.7 (21.2-33-4)	n.s.
HSPCs [x10^6/L]*	**38.6 (20.8-112.4)**	**417.4 (231.5-618.5)**	**<0.001**	466.9 (240.4 – 607.9)	329.4 (205.4-630.9)	n.s.
HSPCs of cells [%]*	**0.9 (0.6-4.6)**	**5.5 (4.6-7.8)**	**0.001**	5.3 (4.5-8.3)	5.6 (4.6-7.1)	n.s.
HSPCs of MNC [%]*	**3.0 (1.8-12.8)**	**41.3 (34.4-53.3)**	**<0.0001**	40.5 (32.6-51.8)	43.2 (36.7-55.1)	n.s.
CD39+ HSPCs of MNC [%]*	**1.3 (0.5-6.6)**	**33.3 (20.0-43.4)**	**<0.0001**	30.4 (19.7-43.2)	35.7 (21.3-42.9)	n.s.
CD39+HSPCs [x10^6/L]*	**14.4 (7.3-80.5)**	**289.6 (160.4 471.2)**	**<0.0001**	273.5 (180.7-465.4)	289.6 (122.6-486.8)	n.s.
CD73+ HSPC of MNCs [%]*	**0.3 (0.1-1.0)**	**1.6 (0.6-3.4)**	**0.0008**	1.6 (0.6-3.4)	1.4 (0.7-6.2)	n.s.
CD73+ HSPCs [x10^6/L]*	**4.3 (1.5-8.8)**	**14.2 (6.3-38.3)**	**0.005**	16.6 (7.2-37.4)	13.6 (6.0-38.8)	n.s.
G-CSFR+ HSPCs [x10^6/L]*	**12.6 (7.4-62.4)**	**153.2 (87.9-287.6)**	**<0.0001**	153.2 (89.8-278.7)	149.6 (80.7-301.9)	n.s.
G-CSF+ HSPCs of MNC [%]*	**1.1 (0.5-6.1)**	**18.0 (11.5-25.2)**	**<0.0001**	17.7 (10.8-23.6)	18.0 (13.8-25.9)	n.s.
pDC [x10^6/L]*	**1.95 (1.43-3.00)**	**5.82 (2.59-8.46)**	**0.01**	5.13 (2.34-8.34)	6.39 (2.72-9.79)	n.s.
pDC of cells [%]*	0.05 (0.00-0.10)	0.10 (0.00-0.20)	n.s.	0.10 (0.00-0.20)	0.10 (0.00-0.20)	n.s.
mDC [x10^6/L]*	**1.25 (0.15-25.7)**	**26.2 (6.46-97.3)**	**0.004**	32.2 (9.00-113.6)	14.2 (6.19-67.4)	n.s.
mDC of cells [%]*	**0.00 (0.00-0.65)**	**0.60 (0.10-1.70)**	**0.04**	0.70 (0.123-1.88)	0.55 (0.10-1.05)	n.s.
Monocytes [x10^6/L]*	**278.8 (191.4-354.3)**	**497.2 (284.9-702.9)**	**0.009**	509.9 (284.5-694.4)	487.8 (285.5-715.5)	n.s.
Monocytes [%]*	7.6 (6.2-10.3)	6.9 (5.6-9.3)	n.s.	7,0625 5,89375 9,175	6,9125 5,44375 9,03125	n.s.
Classical monocytes [x10^6/L]*	**230.2 (113.9-275.4)**	**421.7 (229.1)-602.8)**	**0.006**	421.0 (242.0-624.0)	396.1 (221.0-591.8)	n.s.
Classical monocytes of monocytes [%]*	**50.7 (36.8-54.5)**	**73.4 (59.4-78.3)**	**<0.0001**	73.9 (59.4-79.5)	73.4 (61.3-76.3)	n.s.
Intermediate monocytes [x10^6/L]*	**5.98 (3.66-10.17)**	**26.0 (11.7-59.8)**	**<0.001**	25.4 (13.1-63.1)	26.9 (10.4-54.8)	n.s.
Intermediate monocytes of monocytes [%]*	**1.5 (0.88-2.78)**	**5.15 (3.0-9.7)**	**0.0004**	5.2 (3.13-9.93)	5.0 (3.0-8.4)	n.s.
Non-classical monocytes [x10^6/L]*	8.6 (4.2-15.5)	4.4 (2.5-11.6)	n.s.	4.1 (2.4-13.2)	5.0 (2.6-9.5)	n.s.
Non-classical monocytes of monocytes [%]*	**2.6 (2.2-3.2)**	**1.0 (0.5-1.7)**	**0.008**	1.0 (0.5-1.8)	1.0 (0.6-1.7)	n.s.
CD64+ monocytes [x10^6/L]*	**167.4 (96.3-197.8)**	**342.4 (190.5-537.1)**	**0.002**	346.0 (178.1-581.7)	341.2 (204.2-512.7)	n.s.
CD64+ of monocytes [%]*	**75.5 (63.0-78.6)**	**84.9 (73.6-91.4)**	**0.01**	83.2 (72.4-91.1)	88.7 (77.2-91.6)	n.s.
CD32+ monocytes [x10^6/L]*	12.4 (6.94-17.4)	16.9 (6.62-40.5)	n.s.	20.5 (7.92-38.9)	14.2 (4.73-41.0)	n.s.
CD32+ of monocytes [%]*	6.5 (2.8-12.3)	4.15 (2.0-7.8)	n.s.	4.7 (2.2-9.8)	3.4 (1.4-5.6)	n.s.
Granulocytes [x10^6/L]*	**1700.6 (1506.9-2632.4)**	**4584.4 (2601.1-7946.7)**	**<0.001**	4552.5 (2689.8-9161.1)	4584.4 (2774.1-7675.2)	n.s.

Granulocytes of cells [%]*	**61.8 (54.7-62.9)**	**85.2 (79.0-88.5)**	**<0.0001**	85.2 (79.4-88.1)	83.9 (78.6-89.1)	n.s.
Neutrophils [x10⁶/L]*	**1652.1 (1423.9-2218.8)**	**4334.6 (2518.5-7446.9)**	**<0.001**	4283.2 (2617.5-8560.1)	4427.7 (2600.2-7418.8)	n.s.
Neutrophils of granulocytes [%]*	94.6 (90.6-97.5)	95.8 (92.1-97.5)	n.s.	95.7 (91.9-98.0)	95.8 (92.5-97.3)	n.s.
CD39+ of neutrophiles [%]*	16.0 (9.8-25.4)	33.2 (15.7-48.4)	n.s.	30.8 (18.3-43.5)	37.0 (12.2-54.5)	n.s.
CD73+ of neutrophiles [%]*	0.1 (0.0-0.2)	0.1 (0.0-0.1)	n.s.	0.1 (0.0-0.1)	0.1 (0.0-0.1)	n.s.
Eosinophils [x10⁶/L]*	63.0 (37.7-137.0)	129.3 (63.9-228.0)	n.s.	115.0 (66.8-218.1)	139.7 (62.1-224.0)	n.s.
Eosinophils of granulocytes [%]*	5.0 (2.1-8.5)	2.8 (1.2-5.1)	n.s.	2.7 (0.98-5.2)	2.8 (1.5-4.8)	n.s.
Lymphocytes [x10⁶/L]*	**1013.1 (687.0-1180.7)**	**353.9 (203.7-632.0)**	**0.0001**	353.2 (209.8-643.1)	353.9 (209.8-568.1)	n.s.
Lymphocytes of cells [%]*	**26.3 (23.0-28.3)**	**5.9 (4.0-9.7)**	**<0.0001**	5.9 (4.7-9.3)	5.9 (2.9-9.9)	n.s.
CD39+ of lymphocytes [%]*	12.5 (9.1-15.0)	14.6 (10.4-21.6)	n.s.	15.0 (10.6-19.6)	14.3 (10.4-23.7)	n.s.
CD32 of lymphocytes [%]*	13.9 (12.2-16.0)	13.3 (8.8-21.1)	n.s.	12.2 (8.4-17.1)	14.8 (9.6-27.7)	n.s.
T cells [x10⁶/L]*	**780.6 (463.7-954.3)**	**252.7 (138.2-450.7)**	**<0.001**	277.3 (148.9-474.4)	233.5 (130.9-426.3)	n.s.
T cells of cells [%]*	**21.8 (19.7-26.5)**	**4.9 (2.5-7.0)**	**<0.0001**	5.0 (2.8-6.8)	4.7 (2.0-7.5)	n.s.
CD4+ T cells [x10⁶/L]*	**404.6 (275.0-554.9)**	**178.1 (77.1-353.4)**	**0.01**	214.1 (69.3-392.0)	169.2 (113.8-308.1)	n.s.
CD4+ of T cells [%]*	**55.6 (51.2-60.4)**	**75.1 (64.7-80.1)**	**0.003**	72.6 (61.7-79.9)	75.3 (68.4-80.4)	n.s.
CD8+ T cells [x10⁶/L]*	**221.1 (121.3-300.2)**	**38.9 (16.4-69.4)**	**<0.001**	44.4 (20.4-77.7)	30.7 (9.47-50.8)	n.s.
CD8+ of T cells [%]*	**36.0 (30.0-38,9)**	**13.6 (9.2-19.3)**	**<0.0001**	15.6 (10.6-23.1)	10.8 (8.7-15.4)	n.s.
CD4/CD8 ratio	**1.56 (1.26-2.08)**	**5.55 (2.83-8.53)**	**<0.001**	4.74 (2.36-7.51)	6.74 (5.09-8.81)	n.s.
CD4+CD25+ T cells [x10⁶/L]*	20.6 (11.5-38.2)	14.1 (7.20-27.8)	n.s.	14.1 (6.18-26.8)	12.1 (7.26-28.4)	n.s.
CD25+ of CD4+T cells [%]*	7.0 (3.3-10.0)	9.1 (5.9-12.1)	n.s.	10.0 (6.2-11.9)	8.1 (5.6-13.5)	n.s.
CD8+CD25+T cells [x10⁶/L]*	**4.83 (3.40-5.68)**	**1.74 (0.91-3.45)**	**0.007**	2.19 (1.18-4.38)	1.13 (0.74-3.17)	n.s.
CD25+ of CD8+T cells [%]*	**3.1 (1.5-4.2)**	**6.9 (3.5-10.1)**	**0.008**	7.0 (3.6-10.6)	6.9 (3.6-9.6)	n.s.
CD39+ T cells [x10⁶/L]*	29.7 (22.0-63.3)	26.8 (13.3-64.5)	n.s.	33.6 (19.9-71.6)	22.6 (11.8-49.3)	n.s.
CD39+ of T cells [%]*	**6.25 (3.5-8.0)**	**12.2 (8.6-18.8)**	**0.0009**	12.8 (8.5-20.1)	10.3 (9.0-18.2)	n.s.
Tregs [x10⁶/L]*	**31.3 (18.4-45.4)**	**12.1 (7.14-24.5)**	**0.005**	10.6 (7.5-24.0)	13.6 (6.13-26.3)	n.s.
Tregs of T cells [%]*	4.9 (4.0-5.7)	4.6 (4.0-7.0)	n.s.	4.6 (3.9-5.9)	4.9 (4.0-8.4)	n.s.
CD39+Tregs [x10⁶/L]*	10.5 (6.02-12.5)	6.88 (3.27-14.0)	n.s.	6.89 (2.95-13.18)	6.18 (3.51-14.98)	n.s.
CD39+ of Tregs [%]*	**38.0 (19.0-50.0)**	**60.4 (44.7-70.9)**	**0.007**	60.3 (44.7-71.9)	61.0 (45.4-67.3)	n.s.
NKT cells [x10⁶/L]*	**18.0 (15.0- 30.5)**	**8.91 (5.13-18.0)**	**0.01**	10.0 (5.64-19.2)	8.40 (3.89-141)	n.s.
NKT cells of T cells [%]*	4.0 (2.4-4.7)	4.2 (1.7-8.3)	n.s.	4.3 (1.7-9.0)	4.2 (1.7-7.7)	n.s.
CD39+ NKT cells [x10⁶/L]*	0.99 (0.52-1.47)	1.16 (0.49-2.34)	n.s.	1.25 (0.86-2.42)	0.90 (0.36-2.21)	n.s.
CD39+ of NKT cells [%]*	**3.1 (1.7-11.4)**	**15.5 (7.0-24.1)**	**0.003**	14.2 (7.1-20.5)	18.3 (7.4-28.8)	n.s.
CD39+NKT of Tcells [%]*	**0.2 (0.1-0.2)**	**0.5 (0.2-1.2)**	**0.004**	0.5 (0.2-1.1)	0.5 (0.2-1.3)	n.s.
NK cells [x10⁶/L]*	62.4 (44.3-78.2)	44.0 (20.2-68.3)	n.s.	38.7 (17.4-67.2)	44.9 (21.7-67.5)	n.s.

NK cells of cells [%]*	**2.3 (1.7-4.2)**	**0.7 (0.4-1.4)**	**0.0003**	0.7 (0.4-1.4)	0.7 (0.4-1.3)	n.s.
CD39+ NK cells [x10⁶/L]*	**1.85 (0.83-4.63)**	**5.47 (2.22-11.5)**	**0.02**	5.47 (2.39-11.0)	5.13 (1.95-11.7)	n.s.
CD39+ of NK cells [%]*	**2.7 (1.6-3.9)**	**14.6 (7.0-28.8)**	**<0.0001**	14.8 (7.3-34.6)	14.2 (6.9-25.0)	n.s.
B cells [x10⁶/L]*	**98.4 (77.3-152.1)**	**30.5 (16.7-64.1)**	**<0.001**	26.5 (16.6-60.2)	40.1 (21.9-76.6)	n.s.
B cells of cells [%]*	**3.4 (2.8-3.8)**	**0.7 (0.3-1.1)**	**<0.0001**	0.6 (0.3-1.0)	0.8 (0.4-1.3)	n.s.
CD39+B cells [x10⁶/L]*	**93.1 (71.8-136.2)**	**27.36 (16.12-61.54)**	**<0.001**	23.14 (15.45-57.07)	37.11 (21.22-68.05)	n.s.
CD39+ of B cells [%]*	93.4 (88.2-94.5)	95.1 (90.8-97.1)	n.s.	95.4 (91.9-97.6)	94.7 (90.0-96.6)	n.s.
CD39+ B cells of cells [%]*	**1.5 (0.8-2.1)**	**0.6 (0.3-1.1)**	**0.02**	0.5 (0.3-0.9)	0.8 (0.3-1.3)	n.s.

*Data are presented as median value (IQR); underlined parameters were further analyzed in multivariate Cox analysis; bold values show significant differences between controls and ACLF patients

IL=interleukin, IFN-g=interferon-γ, TNF-a=tumor necrosis factor-α, CXCL12/SDF-1a= stromal cell-derived factor-α, VEGF-A=vascular endothelial growth factor-A, SCF= stem cell factor, HGF= hepatocyte growth factor, MNC= mononuclear cell, pDC = plasmacytoid dendritic cells, mDC = myeloid dendritic cell, NKT cells = natural killer T cells, NK cells = natural killer cells; n.s=not significant (p>0.05; Mann-Whitney)

Table S2: Clinical characteristics of the study population.

Parameter	*All patients (n=79)*	*G-CSF (n=40)*	*SMT (n= 39)*	*p*
Age [years]*	55.0 (51.5-61.0)	54.5 (51.0-59.0)	56.0 (52.0-63.5)	n.s.
Sex [n], male/female	54/25	24/16	30/9	n.s.
BMI*	29.4 (25.1-33.1)	29.4 (26.2-31.2)	28.7 (24.7-33.5)	n.s.
Ascites [n], y/n	77/2	39/1	38/1	n.s.
Albumin [g/L]*	31.0 (27.6-35.7)	30.9 (27.3-35.8)	32.0 (27.7-34.2)	n.s.
Alpha-fetoprotein [ng/mL]*	2.1 (1.0-3.1)	2.1 (1.4-2.9)	2.1 (1.0-3.7)	n.s.
ALT [µkat/L]*	0.68 (0.37-1.20)	0.68 (0.37-1.03)	0.70 (0.38-1.25)	n.s.
AP [µkat/L]*	2.2 (1.6-2.8)	2.2 (1.8-2.6)	2.2 (1.3-2.9)	n.s.
AST [µkat/L]*	1.33 (0.82-1.97)	1.33 (0.93-1.84)	1.28 (0.79-2.39)	n.s.
Basophiles [/µL]*	30.2 (11.0-61.8)	31.6 (12.5-62.7)	30 (10.4-60.0)	n.s.
Bilirubin [µmol/L]*	342.5 (86.4-488.3)	365.2 (93-486.8)	316.8(81.6-502.2)	n.s.
CRP [mg/L]*	32.8 (16.8-57.2)	31.7 (17.2-59.4)	38.3 (17.1-49.3)	n.s.
Eosinophils[/µL]*	165.0 (90.0-326.0)	161.3 (90.0-284.0)	172.0 (90.0-377.4)	n.s.
Erythrocytes [x10^6/µL]*	2.67 (2.40-3.03)	2.67 (2.45-2.96)	2.70 (2.40-3.26)	n.s.
GGT [µkat/L]*	1.49 (0.59-3.54)	1.42 (0.62-3.44)	1.71 (0.55-3.43)	n.s.
HB [mmol/L]*	5.4 (5.0-6.4)	5.7 (5.0-6.2)	5.3 (4.8-6.6)	n.s.
Hematocrit [L/L]*	0.25 (0.23-0.29)	0.25 (0.24-0.29)	0.26 (0.23-0.29)	n.s.
Creatinine [µmol/L]*	188.8 (104.75-270)	185.8 (89.7-242.8)	195 (115.3-279.8)	n.s.
Leukocytes [x10^9 L]*	10.03 (6.70-14.75)	10.90 (7.35-15.02)	8.70 (5.63-14.25)	n.s.
Monocytes [x10^9/L]*	0.83 (0.50-1.10)	0.90 (0.57-1.08)	0.77 (0.39-1.10)	n.s.
Neutrophils [x10^9/L]*	7.04 (4.29-11.63)	7.75 (4.95-11.67)	6.64 (4.14-11.52)	n.s.
Potassium [mmol/L]*	3.9 (3.5-4.3)	3.9 (3.5-4.21)	3.92 (3.59-4.39)	n.s.
Procalcitonin [ng/mL]*	0.82 (0.47-1.76)	0.68 (0.46-1.45)	1.12 (0.56-1.78)	n.s.
Sodium [mmol/L]*	135.0 (131.0-138.0)	134.6 (130.8-137.3)	135 (131.3-138.8)	n.s.
Thrombocytes [x10^9/L]*	71.0 (45.5-124.5)	76.0 (50.0-149.5)	63.0 (32.0-102.0)	n.s.
Urea [mmol/L]*	18.7 (9.4-22.3)	16.2 (8.5-21.0)	19.5 (14.8-26.7)	n.s.
ACLF grade [n], 1/2/3	35/30/13	17/18/5	18/12/8	n.s.
MELD score*	24.83 (20.25-28.10)	24.9 (21.9-27.7)	24.6 (20.1-29.4)	n.s.
Child Pugh score [n], A/B/C	0/14/64	0/9/31	0/5/33	n.s.
CLIF-C OF*	10 (9-12)	10 (9-11)	11 (9-12)	n.s.
HE [n], y/n	59/20	29/11	30/9	n.s.
Death/Transplant within 30d [n]	29/8	14/3	15/5	
Death/Transplant within 90d [n]	40/9	17/4	23/5	
Death/Transplant within 360d [n]	46/13	21/5	25/8	

*Data are presented as median value (IQR)

ALT=alanine aminotransferase, AP=alkaline phosphatase, AST=aspartate transaminase, CRP=C-reactive protein, GGT=γ-gutamyltransferase, HB=haemoglobin, CLIF-C OF score = CLIF-C organ failure score, HE = Hepatic encephalopathy; n.s=not significant (p>0.05; Mann-Whitney or Fischer)

Table S3: Baseline characteristics of subgroups

	Sub-study (n=79)	With baseline biomarkers (n=60)	Without baseline biomarkers (n=19)	Entire GRAFT cohort (n=176)*
Age (years - mean±SD)	55.0 ± 9.6	55.3 ± 9.8	53.9 ± 9.4	54.7 ± 9.9
Gender (f/m)	25 (31.6%)/ 54 (68.4%)	20 (33.3%)/ 40(66.7%)	5 (26.3%)/ 14(73.7%)	65 (36.9%)/ 111 (63.1%)
BMI (kg/m^2 -mean±SD)	29.1 ± 5.6	29.3 ± 5.9	28.4 ± 4.7	28.8 ± 5.2
Sepsis	5 (6.3%)	3 (5.0%)	2 (10.5%)	8 (4.3%)
Bacterial infection at baseline	39 (49.4%)	28 (46.7%)	11 (57.8%)	95 (54.5%)
**Disease severity**				
ACLF grade 1	38 (48.1%)	25 (41.7%)	13 (68.4%)	84 (47.7%)
ACLF grade 2	30 (38.0%)	26 (43.3%)	4 (21.1%)	65 (36.9%)
ACLF grade 3	11 (13.9%)	9 (15.0%)	2 (10.5%)	27 (15.3)
MELD score (mean±SD)	24.8 ± 6.1	25.2 ± 6.2	23.6 ± 5.9	24.4 ± 6.3
CLIF-C OF (mean±SD)	10.7 ± 2.0	10.9 ± 2.0	10.2 ± 2,1	10.4 ± 1.9
**Outcome**				
Number of deaths	46 (58.2%)	35 (58.3%)	11 (57.9%)	109 (61.9%)
Number of OLT by 90 days	9 (11.4%)	8 (13.3%)	1 (5.3%)	14 (8.0%)

*Further characteristics of the GRAFT cohort are published in supplementary material of the GRAFT study.(1)

Table S4: Cox regression analysis for variables associated with TFS in patients with ACLF

	univariate		*multivariate*	
	**HR (95%CI for HR)**	**p value**	**HR (95%CI for HR)**	**p value**
%HSPCs of MNC	**2.1(1.3-3.3)**	**0.0015**		
%CD39+ HSPCs of MNC	**2.3(1.4-3.8)**	**0.0015**		
CD73+ HSPCs /µL	**1(1-1.1)**	**0.0006**		
%CD73+ HSPCs of MNC	**3.2(1.7-6.3)**	**0.0006**	**1.186 (1.039-1.354)**	**0.0113**
%G-CSFR+ HSPCs of MNC	**2.8(1.4-5.6)**	**0.0026**		
%CD73+ of neutrophils	**3.6(1.2-11)**	**0.024**		
%CD39+ of lymphocytes	**1(1-1.1)**	**0.0085**		
%CD64+ of monocytes	**0.97(0.95-1)**	**0.041**		
%CD32+ of lymphocytes	**1(1-1.1)**	**0.033**		
B cells/µL	**1(1-1)**	**0.0046**		
%B cells	**1.6(1.1-2.3)**	**0.012**		
CD39+ B cells/µL	**1(1-1)**	**0.002**		
%CD39+ of B cells	**1(1-1)**	**0.049**		
%CD39+ B cells	**1.7(1.2-2.5)**	**0.0064**		
CD39+NKT cells/µL	**1.1(1.1-1.2)**	**0.0016**	**0.627(0.416-0.947)**	**0.0266**
%CD39+NKT of T cells	**1.5(1.2-1.9)**	**0.0004**		
TNF-a	**1.1(1-1.2)**	**0.0014**	**1.1081.018-1.205)**	**0.0170**
VEGF-A	**0.99(0.98-1)**	**0.032**	**0.979(0.952-0.996)**	**0.0174**

VEGF-A=vascular endothelial growth factor, TNF-a=tumor necrosis factor-α, MNC= mononuclear cell

Table S5: Cox regression analysis of clinical parameters regarding G-CSF treatment in patients with ACLF

	univariate			
	**G-CSF**		**SMT**	
	**HR (95%CI for HR)**	**p value**	**HR (95%CI for HR)**	**p value**
Albumin	0.93 (0.38-2.2)	0.86	1.3 (0.5-3.4)	0.59
Alpha-fetoprotein	0.82 (0.6-1.1)	0.2	1 (0.94-1.2)	0.44
ALT	0.84 (0.44-1.6)	0.6	1 (0.86-1.2)	1
AP	1.4 (0.83-2.3)	0.22	**1.2 (1-1.4)**	**0.023**
AST	1 (0.81-1.3)	0.84	1.1 (0.93-1.3)	0.27
Basophiles	1 (0.99-1)	0.59	1 (0.99-1)	0.28
Bilirubin	1 (1-1)	0.24	1(1-1)	0.07
CRP	0.99 (0.97-1)	0.17	1 (0.99-1)	0.27
Eosinophils	1 (1-1)	0.27	1 (1-1)	0.055
Erythrocytes	1 (1-1)	0.4	1 (1-1)	0.55
GGT	0.86 (0.65-1.1)	0.31	**1.1 (1-1.2)**	**0.007**
HB	0.82 (0.52-1.3)	0.41	0.87 (0.68-1.1)	0.29
Creatinine	1 (1-1)	0.53	1 (1-1)	0.26
Leukocytes	1 (1-1)	0.98	1 (1-1)	0.26
Monocytes	1 (1-1)	0.34	1 (1-1)	0.11
Neutrophils	1 (1-1)	0.68	1 (1-1)	0.54
Potassium	1.7 (0.92-3.1)	0.091	1.4 (0.9-2.1)	0.14
Procalcitonin	1.2 (0.7-1.9)	0.56	**1.2 (1-1.5)**	**0.046**
Sodium	0.93 (0.85-1)	0.073	1 (0.95-1.1)	0.96
Thrombocytes	1 (1-1)	0.2	1 (1-1)	0.36
Urea	1 (0.98-1.1)	0.42	1 (0.99-1.1)	0.15
ACLF grade	**1.9 (1-3.6)**	**0.05**	**1.6 (1-2.6)**	**0.035**
MELD score	**1.1 (1-1.2)**	**0.012**	**1.1 (1-1.1)**	**0.05**
Child Pugh score	1.8 (0.62-5.5)	0.27	2.1 (0.64-7.1)	0.21
CLIF-C OF	**1.5 (1.2-2)**	**0.002**	**1.2 (1-1.5)**	**0.026**
HE	2.6 (0.76-8.9)	0.9	2.6 (0.98-6.9)	0.055

ALT=alanine aminotransferase, AP=alkaline phosphatase, AST=aspartate transaminase, CRP=C-reactive protein, GGT=γ-gutamyltransferase, HB=haemoglobin, CLIF-C OF score = CLIF-C organ failure score, HE = Hepatic encephalopathy

Table S6: Cox regression analysis for variables regarding G-CSF treatment in patients with ACLF

	univariate				*multivariate*	
	G-CSF		*SMT*			
	HR (95%CI for HR)	p	HR (95%CI for HR)	p	HR (95%CI for HR)	p
%CD39+ MNC	**1.1(1-1.2)**	**0.045**	0.98(0.88-1.1)	0.76		
%HSPCs of MNC	**2.4(1.3-4.4)**	**0.007**	1.7(0.85-3.4)	0.13		
%CD39+ HSPCs of MNC	**2.6(1.3-5.1)**	**0.007**	1.8(0.8-4)	0.16		
**% G-CSFR+ *MNC***	**1.3(1-1.7)**	**0.029**	1(0.85-1.2)	0.96		
**% G-CSFR+ *HSPCs of MNC***	**3.4(1.3-9.2)**	**0.013**	2.1(0.76-5.6)	0.16		
%HLADR++ MNC	**1.5(1-2.4)**	**0.048**	0.7(0.44-1.1)	0.13		
%CD32+ MNC	**1.9(1.2-2.8)**	**0.003**	0.75(0.52-1.1)	0.13		
%CD11b+ MNC	**1.1(1-1.2)**	**0.03**	0.98(0.9-1.1)	0.6		
%CD73+ of neutrophils	**4.7(1.2-18)**	**0.026**	1.8(0.24-14)	0.56		
%HLADR++of eosinophils	**1.2(1.1-1.4)**	**0.008**	1(0.96-1.1)	0.31		
%Monocytes	***1.1(1-1.1)***	**0.044**	0.98(0.9-1.1)	0.58		
%CD39+ Monocytes	***1.1(1-1.2)***	**0.026**	0.98(0.87-1.1)	0.75		
HLADR++ Monocytes µL	***1(1-1)***	**0.007**	1(0.99-1)	0.74		
%HLADR++ of Monocytes	***1.1(1-1.1)***	**0.024**	0.98(0.95-1)	0.34		
%HLADR++Monocytes	**1.4(1.1-1.9)**	**0.005**	0.85(0.67-1.1)	0.2		
%CD64+ of monocytes	**0.96(0.92-1)**	**0.042**	0.98(0.95-1)	0.39	**0.96(0.93-0.99)**	**0.022**
%HLADR++ of lymphocytes	***1.1(1-1.1)***	**0.006**	1(0.93-1.1)	0.88		
%CD32+ of lymphocytes	***1.1(1-1.1)***	**0.003**	1(0.96-1)	0.97		
%CD39+ of lymphocytes	***1.1(1-1.1)***	**0.021**	1(0.99-1.1)	0.19	**1.05(1.01-1.09)**	**0.008**
B cells/µL	**1(1-1)**	**0.027**	1(1-1)	0.11		
% B cells	**1.8(1.1-2.9)**	**0.013**	1.1(0.62-2.1)	0.68		
CD39+B cells/µL	***1(1-1)***	**0.016**	*1(1-1)*	0.098		
%CD39+B cells	**1.9(1.2-3.1)**	**0.011**	1.2(0.6-2.5)	0.58		
CD39+ NKT cells/µL	**1.2(1-1.3)**	**0.005**	1.1(0.97-1.3)	0.12		

Table S7: Baseline characteristics of patients in clusters 1-2 and 3

Parameter	*Clusters 1-2 (n=29)*	*Cluster 3 (n=31)*	*p*
GCSF treatment[n], y/n	13/16	20/11	n.s.
Clinical Data			
*Age [years]**	57.0 (52.0-64.0)	54.0 (52.0-58.0)	n.s.
Sex [n], male/female	16/13	24/7	n.s.
*BMI**	29.9 (24.7-34.2)	29.2 (25.9-31.5)	n.s.
Ascites [n], y/n	28/1	30/1	n.s.
*Albumin [g/L]**	33.1 (27.9-36.0)	30.5 (25.7-37.1)	n.s.
*Alpha-fetoprotein [ng/mL]**	2.3 (1.6-5.9)	2.1 (1.1-2.9)	n.s.
*ALT [µkat/L]**	0.65 (0.40-1.08)	0.78 (0.53 -1.25)	n.s.
*AP [µkat/L]**	2.5 (1.7-3.0)	2.2 (1.8-2.8)	n.s.
*AST [µkat/L]**	1.14 (0.78-1.63)	1.51 (1.13-2.10)	n.s.
*Basophiles [/µL]**	32.9 (10.0-67.6)	23.7 (11.3-69.6)	n.s.
*Bilirubin [µmol/L]**	363.4(78.9-528.4)	383.6(316.8-514.5)	n.s.
*CRP [mg/L]**	25.3 (9.3-44.4)	35.1 (20.7-64.7)	n.s.
*Eosinophils[/µL]**	157.5 (40.0-279.9)	195.5 (111.5-412.1)	n.s.
*Erythrocytes [x10^6/µL]**	2.67 (2.45-2.87)	2.7 (2.45-3.17)	n.s.
*GGT [µkat/L]**	1.65 (0.69-3.08)	2.0 (1.01-3.88)	n.s.
*HB [mmol/L]**	5.1 (5.0-6.3)	5.8 (5.1-6.6)	n.s.
*Hematocrit [L/L]**	0.25 (0.23-0.28)	0.26 (0.24-0.29)	n.s.
*Creatinine [µmol/L]**	201.5 (113.0-265.0)	185.0 (98.5-255.5)	n.s.
*Leukocytes [x10^9 L]**	8.83 (6.43-15.50)	11.10 (7.39-14.05)	n.s.
*Monocytes [x10^9/L]**	0.92 (0.61-1.10)	0.88 (0.52-1.21)	n.s.
*Neutrophils [x10^9/L]**	6.65 (4.05-14.50)	7.93 (5.67-10.86)	n.s.
*Potassium [mmol/L]**	4.01 (3.58-4.24)	3.9 (3.6-4.26)	n.s.
*Procalcitonin [ng/mL]**	0.73 (0.46-1.59)	1.01 (0.62-2.14)	n.s.
*Sodium [mmol/L]**	133.8 (129.7-137.0)	134.2 (131.5-139.1)	n.s.
*Thrombocytes [x10^9/L]**	82.0 (58.0-116.0)	64.0 (41.5-128.5)	n.s.
*Urea [mmol/L]**	18.6 (14.1-21.5)	19.3 (8.6-23.1)	n.s.
ACLF grade [n], 1/2/3	12/13/4	13/13/5	n.s.
*MELD score**	24.7 (20.6-28.0)	25.4 (21.7-30.3)	n.s.
Child Pugh score [n], A/B/C	0/5/23	0/6/25	n.s.
*CLIF-C OF**	10 (9-11)	11 (10-12)	n.s.
HE	24/5	21/10	n.s.
Sepsis[n], y/n	2/27	1/29	n.s.
Bacterial infection[n], y/n	14/15	14/16	n.s.
Death/Transplant within 30d [n]	11/6	11/1	
Death/Transplant within 90d [n]	14/7	15/2	
Cytokines			
*IL-1b [pg/mL]**	1.18 (0.69-3.51)	2.98 (1.71-3.77)	n.s.
*IL-6 [pg/mL]**	21.44 (9.61-33.27)	20.03 (10.47-32.80)	n.s.
*CXCL8/IL-8 [pg/mL]**	22.68 (12.53-69.52)	50.61 (24.55-128.13)	n.s.
*IFN-g [pg/mL]**	8.45 (4.97-14.07)	9.54 (4.97-13.87)	n.s.
*IL-10 [pg/mL]**	2.09 (0.85-4.48)	1.47 (1.19-2.12)	n.s.

IL-4 [pg/mL]*	26.21 (10.79-37.67)	30.36 (20.43-52.25)	n.s.
TNF-a [pg/mL]*	6.02 (2.91-8.66)	5.97 (3.60-7.49)	n.s.
CXCL12/SDF-1a [pg/mL]*	923.4 (588.3-1376.2)	888.7 (518.4-1087.4)	n.s.
VEGF-A [pg/mL]*	**24.87 (15.48-38.55)**	**35.47 (25.63-57.70)**	**0.03**
SCF [pg/mL]*	130.8 (89.8-187.6)	145.2 (108.4-188.3)	n.s.
Collagen IVa [pg/mL]*	**2222.31 (1573.6-4494.8)**	**4743.1 (2699.9-9789.9)**	**0.009**
HGF [pg/mL]*	**1122.3 (670.0-3032.5)**	**3568.0 (1287-5231.2)**	**0.02**

*Data are presented as median value (IQR)

ALT=alanine aminotransferase, AP=alkaline phosphatase, AST=aspartate transaminase, CRP=C-reactive protein, GGT=γ-gutamyltransferase, HB=haemoglobin, CLIF-C OF score=CLIF-C organ failure score, H =Hepatic encephalopathy; n.s=not significant (bold-p>0.05; Mann-Whitney)

Table S8: Biomarker characteristics in G-CSF-treated patients at baseline and V2

Parameter									
Cytokines	**B (n=34)**	**V2 (n=32)**	**p**	**B Clusters 1-2 (n=14)**	**V2 Clusters 1-2 (n=13)**	**p**	**B Cluster 3 (n=20)**	**V2 Cluster 3 (n=19)**	**p**
IL-1b [pg/mL]*	3.3 (1.5-5.4)	1.7 (0.8-3.4)	n.s.	0.9 (0.7-1.5)	1.2 (0.8-2.6)	n.s.	3.5 (2.9-5.6)	2.8 (1.8-3.9)	n.s.
IL-6 [pg/mL]*	19.6 (10.0-26.8)	18.3 (10.6-40.8)	n.s.	**21.5 (11.0-26.5)**	**28.7 (17.7-48.7)**	**0.02**	19.5 (10.2-28.0)	13.1 (5.8-38.2)	n.s.
CXCL8/IL-8 [pg/mL]*	**40.1 (14.2-113.3)**	**16.1 (6.2-47.1)**	**0.006**	21.3 (12.7-113.3)	27.8 (3.7-69.5)	n.s.	**39.2 (21.8-59.7)**	**12.6 (6.3-19.7)**	**0.03**
IFN-g [pg/mL]*	10.4 (7.1-1 5.0)	10.0 (4.4-13.4)	n.s.	8.4 (3.8-12.4)	7.8 (9.3-3.8)	n.s.	10.8 (7.8-14.6))	9.5 (4.4-13.7)	n.s.
IL-10 [pg/mL]*	1.4 (1.0-2.5)	1.7 (0.8-4.2)	n.s.	1.7 (1.0-4.1)	3.2 (1.2-5.8)	n.s.	1.4 (1.0-2.1)	0.9 (0.6-1.8)	n.s.
IL-4 [pg/mL]*	46.1 (20.6-74.1)	42.5 (11.9-61.7)	n.s.	24.8 (14.2-63.5)	12.4 (8.2-56.6)	n.s.	46.1 (23.9-67.8)	42.5 (15.7-54.5)	n.s.
TNF-a [pg/mL]*	6.0 (3.3-7.7)	4.7 (3.3-8.2)	n.s.	**3.4 (2.8-6.6)**	**4.0 (2.1-8.5)**	**0.02**	6.0 (3.7-7.7)	5.9 (3.4-7.3)	n.s.
CXCL12/SDF-1a [pg/mL]*	943.3 (615.9-1250.0)	983.1 (683.7-1455.5)	n.s.	1168.8 (794.0-1445.0)	1254.1 (1049.0-2276.4)	n.s.	915.0 (421.0-1046.8)	619.7 (685.4-1154.7)	n.s.
VEGF-A [pg/mL]*	33.0 (23.3-59.7)	39.5 (18.3-70.2)	n.s.	28.9 (13.4-46.6)	17.2 (10.6-46.3)	n.s.	35.5 (26.5-53.8)	46.7 (27.7-71.0)	n.s.
SCF [pg/mL]*	138.7 (104.9-172.0)	141.3 (89.8-180.6)	n.s.	112.3 (87.5-163.3)	140.6 (65.4-263.7)	n.s.	144.1 (132.3-172.9)	153.6 (128.8-186.8)	n.s.
Collagen IVa [pg/mL]*	4093.3 (2016.8-8911.3)	3801.1 (1896.1-7468.9)	n.s.	3362.8 (1623.9-6652.0)	2954.7 (1583.7-4430.1)	n.s.	4436.7 (2694.9-11040.4)	3879.0 (11884.3-7404.1)	n.s.
HGF [pg/mL]*	**2241.8 (868.2-5213.9)**	**3284.1 (1461.2-6062.5)**	**0.04**	**1442.8 (742.2-4638.9)**	**3068.9 (1226.3-6380.4)**	**0.02**	3229.1 (936.9-5298.5)	3823.1 (2236.1-5686.1)	n.s.
Functional capacity	**B (n=34)**	**V2 (n=32)**	**p**	**B Clusters 1-2 (n=14)**	**V2 Clusters 1-2 (n=13)**	**p**	**B Cluster 3 (n=20)**	**V2 Cluster 3 (n=19)**	**p**
Phagocytizing neutrophils [x10^6/L]*	**3747.8 (2279.8-8726.8)**	**15073.1 (9457.0-26192.3)**	**<0.0001**	**3559.2 (1853.7-4780.1)**	**12096.4 (8733.0-23899.6)**	**0.004**	**4352.7 (2622.7-8745.4)**	**15987.1 (13094.5-26312.1)**	**0.0001**
Phagocytizing neutrophils [%]	93.5 (86.3-95.1)	92.1 (86.1-94.5)	n.s.	92.6 (72.1-95.5)	89.7 (80.4-91.7)	n.s.	93.7 (88.5-94.6)	93.0 (87.3-95.0)	n.s.
Resting oxidizing neutrophils [x10^6/L]*	**52.1 (28.3-92.6)**	**310.7 (128.0-734.6)**	**0.0002**	**59.9 (41.8-131.1)**	**237.9 (130.2-1002.4)**	**0.02**	**38.9 (23.4-86.3)**	**214.1 (86.6-412.0)**	**0.005**
Resting oxidizing neutrophils [%]	1.25 (0.4-2.0)	1.85 (0.83-3,38)	n.s.	1.4 (0.8-2.5)	1.9 (0.9-5.9)	n.s.	0.5 (0.4-2.0)	1.5 (0.5-2.4)	n.s.
Oxidizing neutrophils [x10^6/L]*	**3536.6 (2369.6-6964.0)**	**11801.8 (8591.7-20411.9)**	**<0.0001**	**3545.9 (2344.5-5988.8)**	**11503.2 (8570.1-17864.0)**	**0.004**	**3513.7(2499.2-7173.7)**	**12498.0 (9330.2-21111.9**	**0.0001**
Oxidizing neutrophils [%]	89.5 (78.0-93.8)	79.2 (70.8-92.7)	n.s.	88.2 (80.6-92.8)	78.9 (59.9-84.6)	n.s.	90.8 (74.3-93.8)	78.8 (67.6-96.1)	n.s.
Migrated cells [test/ctrl]	1.24 (0.53-2.01)	0.64 (0.23-1.97)	n.s.	1.24 (0.82-1.86)	0.84 (0.32-1.90)	n.s.	1.21 (0.71-2.23)	0.37 (0.23-1.50)	n.s.
Chemotactically activated cells [%]*	91.5 (22.6-99.3)	92.4 (69.1-99.0)	n.s.	84.1 (17.3-99.1)	85.5 (69.6-98.6)	n.s.	93.8 (36-99.6)	95.4 (43.9-98.9)	n.s.
*Phagocytizing monocytes [x10^6/L]**	**299.0 (167.7-530.6)**	**581.5 (420.8-1031.3)**	**< 0.0001**	**322.4 (233.0-479.9)**	**584.2 (423.5-726.9)**	**0.004**	**321.8 (152.1-551.0)**	**801.6 (488.2-1198.2)**	**0.0002**
Phagocytizing monocytes [%]	73.1 (51.6-81.6)	64.9 (56.5-73.5)	n.s.	71.1 (45.5-80.1)	57.0 (50.8-66.8)	n.s.	73.4 (57.4-81.3)	65.9 (63.3-75.0)	n.s.
*Resting oxidizing monocytes [x10^6/L]**	4.2 (1.2-10.4)	7.9 (2.6-26.8)	n.s.	6.5 (2.0-18.4)	5.9 (4.0-34.2)	n.s.	2.0 (1.1-6.7)	6.1 (2.4-10.5)	n.s.
Resting oxidizing monocytes [%]	1.2 (0.2-3.1)	0.7 (0.2-2.2)	n.s.	1.4 (0.7-2.7)	0.9 (0.2-2.2)	n.s.	0.4 (0.2-3.2)	0.4 (0.2-1.7)	n.s.
*Oxidizing monocytes [x10^6/L]**	**143.4 (63.2-229.3)**	**233.2 (148.3-454.1)**	**0.008**	155.3 (85.9-215.2)	242.3 (147.3-510.8)	n.s.	**138.3 (42.5-221.7)**	**212.3 (140.2-438.9)**	**0.02**
Oxidizing monocytes [%]	41.2 (15.6-52.5)	29.9 (14.5-43.3)	n.s.	31.2 (20.5-46.8)	28.3 (5.5-43.7)	n.s.	43.5 (10.3-55.3)	26.9 (11.9-41.4)	n.s.

Cell populations	B (n=34)	V2 (n=32)	p	B Clusters 1-2 (n=14)	V2 Clusters 1-2 (n=13)	p	B Cluster 3 (n=20)	V2 Cluster 3 (n=19)	p
CD45+ [x10^6/L]*	**6226.9 (3377.6-10360.6)**	**22312.4 (13009.6-34435.7)**	**<0.0001**	**5174.6 (3144.9-8930.1)**	**17264.1 (12941.1-33931.0**	**0.004**	**6620.6 (4171.1-106875.6)**	**23884.9 (16131.7-33671.9)**	**<0.0001**
CD73+ MNC [x10^6/L]*	**85.0 (52.2-161.0)**	**174.5 (87.0-338.2)**	**0.009**	153.0 (55.7-224.3)	155.5 (91.9-194.6)	n.s.	**77.8 (53.2-102.4)**	**274.7 (100.7-359.7)**	**0.006**
CD73+ of MNC [%]*	7.8 (5.2-11.9)	7.7 (5.3-12.0)	n.s.	10.1 (5.8-15.4)	7.1 (6.4-13.1)	n.s.	6.6 (5.1-9.0)	7.9 (5.6-14.3)	n.s.
CD39+MNC [x10^6/L]*	**503.4 (263.8-577.4)**	**984.8 (645.3-1342.5)**	**<0.0001**	568.9 (459.7-604.6)	1031.9 (635.1-1186.3)	n.s.	**395.4 (234.5-552.1)**	**1099.0 (664.4-1466.7)**	**0.0002**
CD39 of MNC [%]*	**49.2 (39.7-52.6)**	**50.3 (43.2-55.8)**	**n.s.**	52.7 (48.1-59.4)	50.1 (40.5-56.3)	n.s.	**43.3 (37.6-50.2)**	**50.3 (44.9-55.2)**	**0.029**
G-CSFR+ MNC [x10^6/L]*	**216.4 (149.2-402.2)**	**473.7 (263.4-886.4)**	**0.0002**	265.6 (175.9-383.8)	381.5 (342.9-593.7)	n.s.	**203.4 (131.8-415.6)**	**528.9 (261.9-1013.3)**	**0.0002**
G-CSFR+ of MNC [%]*	25.6 (19.0-36.4)	24.2 (18.0-29.8)	n.s.	26.4 (19.7-36.4)	23.6 (19.5-28.1)	n.s.	**25.6 (19.1-34.1)**	**24.0 (16.5-30.3)**	**n.s.**
HSPCs [x10^6/L]*	**466.9 (240.4-607.9)**	**847.8 (394.8-1459.1)**	**0.0006**	489.8 (265.9-647.1)	621.4 (210.8-1145.0)	n.s.	**384.2 (204.6-599.9)**	**1177.5 (457.1-1839.0)**	**0.001**
HSPCs of cells [%)*	5.3 (4.5-8.3)	4.5 (2.2-6.6)	n.s.	**8.6 (4.5-10.0)**	**3.9 (2.4-7.6)**	**0.008**	4.9 (4.5-6.2)	4.7 (3.4-6.6)	n.s.
pDC [x10^6/L]*	**5.13 (2.34-8.34)**	**15.4 (6.61-28.7)**	**0.0004**	**6.60 (3.86-8.90)**	**23.3 (15.1-33.9)**	**0.004**	**4.13 (1.67-8.19)**	**13.0 (5.83-26.5)**	**0.003**
pDCs of cells [%]*	0.10 (0.00-0.20)	0.10 (0.05-0.15)	n.s.	0.10 (0.10-0.20)	0.10 (0.10-0.30)	n.s.	0.10 (0.00-0.13)	0.10 (0.00-0.10)	n.s.
mDCs [x10^6/L]*	**32.2 (9.00-113.6)**	**106.3 (36.8-335.0)**	**0.004**	53.5 (12.5-113.5)	219.9 (30.8-314.8)	n.s.	**28.3 (2.30-102.3)**	**105.7 (33.8-391.3)**	**0.02**
mDCs of cells [%]*	0.70 (0.13-1.88)	0.60 (0.25-1.75)	n.s.	1.50 (0.20-2.80)	1.60 (0.20-2.30)	n.s.	0.50 (0.08-1.50))	0.50 (0.23-1.18)	n.s.
Classical monocytes [x10^6/L]*	**421.7 (242.0-623.2)**	**712.7 (543.1-1185.1)**	**<0.0001**	402.2 (266.6-597.3)	958.5 (329.3-1152.1)	n.s.	**447.1 (227.5-637.4)**	**745.3 (574.1-1563.6)**	**0.0004**
Classical monocytes of monocytes [%]*	**73.9 (59.4-79.5)**	**64.6 (55.5-72.1)**	**0.002**	66.2 (55.0-75.8)	60.0 (50.7-64.6)	n.s.	**76.2 (67.2-79.8)**	**68.6 (58.7-73.4)**	**0.02**
Intermediate monocytes [x10^6/L]*	**25.4 (13.1-63.1)**	**79.1 (46.4-188.2)**	**0.0002**	**37.5 (17.4-66.2)**	**154.7 (91.3-186.2)**	**0.04**	**20.1 (11.1-59.7)**	**68.3 (39.7-260.6)**	**0.004**
Intermediate monocytes of monocytes [%]*	5.20 (3.13-9.93)	7.40 (5.45-9.45)	n.s.	8.75 (3.15-11.28)	7.90 (7.20-9.10)	n.s.	4.90 (3.15-6.68)	7.00 (3.73-8.98)	n.s.
Non-classical monocytes [x10^6/L]*	**4.1 (2.4-13.2)**	**26.6 (20.5-70.5)**	**<0.0001**	**4.4 (2.8-13.3)**	**52.9 (24.8-89.3)**	**0.008**	**3.9 (2.3-12.3)**	**24.8 (17.6-57.9)**	**0.0004**
Non-classical monocytes of monocytes [%]*	**1.0 (0.5-1.6)**	**3.1 (1.5-4.9)**	**0.005**	**1.0 (0.4-2.3)**	**3.4 (2.2.-7.3)**	**0.01**	1.0 (0.7-1.6)	2.9 (1.1-4.9)	n.s.
CD64+ monocytes [x10^6/L]*	**346.0 (178.1-581.7)**	**699.9 (498.0-989.6)**	**<0.0001**	**325.8 (168.7-488.7)**	**812.2 (582.3-984.2)**	**0.02**	**369.5 (195.8-606.5)**	**710.4 (538.3-1344.8)**	**0.0003**
CD64+ monocytes of monocytes [%]*	83.2 (72.4-91.1)	84.8 (78.9-90.5)	n.s.	76.4 (67.6-84.9)	89.8 (76.7-94.1)	n.s.	84.7 (80.2-91.6)	83.4 (79.0-88.9)	n.s.
CD32+ monocytes [x10^6/L]*	**20.5 (7.92-38.9)**	**48.5 (16.2-104.8)**	**0.005**	21.0 (10.6-32.8)	69.9 (17.5-92.0)	n.s.	**15.3 (7.7-52.8)**	**54.0 (18.5-112.4)**	**0.008**
CD32+ monocytes of monocytes [%]*	4.65 (2.18-9.75)	5.4 (1.8-10.9)	n.s.	5.95 (2.33-8.0)	5.9 (1.6-10.4)	n.s.	4.15 (2.33-12.65)	5.25 (2.4-12.23)	n.s.
Granulocytes [x10^6/L]*	**4552.5 (2689.8-9161.1)**	**13773.7 (10182.6-27205.8)**	**<0.0001**	**4274.7 (2441.6-6903.8)**	**12844.2 (10226.7-25819.8)**	**0.004**	**4691.8 (3582.7-9369.3)**	**16915.1 (11513.8-28403.4)**	**<0.0001**
Granulocytes of cells [%]*	**85.2 (79.4-88.1)**	**87.5 (84.1-92.8)**	**0.0008**	**78.7 (70.1-85.1)**	**86.6 (79.7-93.5)**	**0.0005**	**85.7 (84.7-88.5)**	**88.1 (84.9-90.6)**	**0.01**
Neutrophils [x10^6/L]*	**4283.2 (2617.5-8560.1)**	**13171.1 (9722.7-26370.0)**	**<0.0001**	**3921.9 (2410.4-6499.5)**	**12542.1 (9692.7-24709.3)**	**0.004**	**4576.0 (3424.0-9117.2)**	**14764.0 (10208.7-27872.0)**	**<0.0001**
Neutrophils of granulocytes [%]*	95.7 (91.9-98.0)	95.6 (92.2-97.4)	n.s.	94.0 (91.3-97.9)	94.9 (93.9-95.7)	n.s.	96.7 (94.3-97.7)	96.2 (90.9-98.0)	n.s.

Eosinophils [x10^6/L]*	**115.0 (66.8-218.1)**	**233.0 (143.9-540.9)**	**0.001**	**106.9 (57.2-155.1)**	**313.6 (211.5-504.2)**	**0.04**	**115.0 (76.7-244.9)**	**231.5 (111.6-630.2)**	**0.02**
Eosinophils of granulocytes [%]*	**2.7 (0.98-5.18)**	**1.9 (0.9-3.2)**	**0.003**	3.1 (0.7-6.9)	2.2 (1.0-3.2)	n.s.	**2.3 (1.2-3.7)**	**1.9 (0.85-2.9)**	**0.02**
T cells [x10^6/L]*	**277.3 (148.9-474.4)**	**472.2 (273.3-797.8)**	**0.004**	164.0 (112.6-498.7)	382.5 (369.5-1060.7)	n.s.	**316.6 (169.8-457.1)**	**573.9 (330.4-753.5)**	**0.01**
T cells of cells[%]*	**5.0 (2.8-6.8)**	**3.0 (1.4-3.9)**	**0.0002**	**5.5 (2.5-8.0)**	**3.0 (1.0-4.3)**	**0.02**	**4.9 (3.8-5.3)**	**3.2 (2.0-3.6)**	**0.005**
CD4+ T cells [x10^6/L]*	**214.1 (69.3-392.0)**	**332.0 (186.3-637.6)**	**0.008**	115.2 (44.3-391.1)	360.3 (269.5-879.9)	n.s.	**237.8 (97.0-380.3)**	**369.9 (190.1-577.9)**	**0.02**
CD4+ of T cells[%]*	72.6 (61.7-79.9)	73.3 (63.9-84.5)		67.1 (43.6-78.7)	73.3 (71.0-85.3)	n.s.	75.2 (64.1-80.6)	73.8 (57.1-83.1)	n.s.
CD8+ T cells [x10^6/L]*	44.4 (20.4-77.7)	59.3 (30.6-121.4)	n.s.	36.0 (17.4-72.7)	57.9 (30.5-112.7)	n.s.	45.8 (25.4-73.3)	83.6 (37.6-135.3)	n.s.
CD8+ of T cells [%]*	**15.6 (10.6-23.1)**	**12.9 (7.7-21.4)**	**0.04**	15.8 (11.1-25.0)	13.7 (8.3-15.9)	n.s.	15.2 (9.8-23.0)	15.4 (8.2-23.0)	n.s.
CD4/CD8 ratio	**4.74 (2.36-7.51)**	**5.82 (2.68-11.29)**	**0.04**	4.42 (2.08-5.98)	5.78 (4.55-10.32)	n.s.	4.90 (2.77-8.57)	4.77 (2.18-10.71)	n.s.
CD4+CD25+ T cells [x10^6/L]*	**14.1 (6.18-26.8)**	**34.9 (12.9-65.1)**	**0.001**	16.4 (8.74-23.1)	53.5 (13.4-74.1)	n.s.	**13.9 (4.95-27.8)**	**39.6 (14.5-53.7)**	**0.01**
CD25+ of CD4+T cells [%]*	10.0 (6.2-11.9)	9.6 (5.2-11.5)	n.s.	11.6 (7.8-16.8)	11.3 (7.9-17.3)	n.s.	7.7 (4.5-10.6)	9.2 (5.2-11.3)	n.s.
CD8+CD25+T cells [x10^6/L]*	2.19 (1.18-4.38)	2.34 (1.04-9.07)	n.s.	2.84 (1.72-10.5)	3.21 (1.37-15.2)	n.s.	2.01 (1.09-3.31)	2.41 (1.05-8.70)	n.s.
CD25+ of CD8+T cells [%]*	7 (3.6-10.6)	5.3 83.2-7.0)	n.s.	8.5 (6.9-13.1)	7.7 (6.9-12.4)	n.s.	4.8 (2.2-7.8)	5.1 (3.4-5.6)	n.s.
CD39+ T cells [x10^6/L]*	**33.6 (19.9-71.6)**	**53.9 (22.8-104.4)**	**0.02**	45.4 (23.7-88.6)	75.4 (29.5-130.8)	n.s.	**27.8 (13.6-55.9)**	**55.3 (23.9-92.1)**	**0.02**
CD39+ of T cells [%]*	12.8 (8.5-20.1)	10.2 (7.2-13.4)	n.s.	17.0 (12.8-28.4)	13.3 (8.3-18.4)	n.s.	9.5 (6.5-14.1)	10.1 (7.2-13.1)	**n.s.**
Tregs [x10^6/L]*	**10.6 (7.5-24.0)**	**26.6 (14.2-54.7)**	**0.003**	9.22 (4.70-21.1)	42.6 (17.0-79.5)	n.s.	**11.9 (8.79-24.3)**	**28.1 (13.5-41.5)**	**0.03**
Tregs+ of T cells [%]*	4.6 (3.9-5.9)	5.2 (4.6-6.7)	n.s.	4.3 (3.7-6.7)	6.2 (5.2-6.8)	n.s.	4.7 (4.1-5.4)	4.9 (4.3-5.8)	n.s.
CD39+Tregs [x10^6/L]*	**6.89 (2.95-13.18)**	**19.5 (6.26-30.8)**	**0.0007**	**6.48 (3.60-11.3)**	**24.3 (7.13-52.8)**	**0.04**	**7.34 (2.39-14.13)**	**20.0 (6.53-28.3)**	**0.01**
CD39+ of Tregs [%]*	60.3 (44.7-71.9)	58.6 (41.1-74.8)	n.s.	63.9 (55.4-79.3)	71.9 (55.8-79.1)	n.s.	59.6 (40.9-68.4)	60.0 (45.9-73.6)	n.s.
NKT cells [x10^6/L]*	**10.0 (5.64-19.2)**	**19.8 (9.89-31.5)**	**0.0009**	**9.06 (5.64-14.0)**	**16.7 (10.1-30.8)**	**0.02**	**12.4 (6.51-24.0)**	**22.0 (9.09-34.0)**	**0.02**
NKT cells of T cells [%]*	4.3 (1.7-9.0)	4.2 (2.5-6.9)	n.s.	**4.0 (1.4-9.6)**	**2.6 (2.1-6.9)**	**0.04**	5.0 (2.0-8.5)	3.9 (2.7-7.0)	n.s.
CD39+ NKT cells [x10^6/L]*	1.25 (0.86-2.42)	1.95 (0.67-2.93)	n.s.	1.31 (1.03-2.57)	2.06 (0.86-2.82)	n.s.	**1.18 (0.57-2.34)**	**2.09 (0.70-3.99)**	**0.04**
CD39+ of NKT cells [%]*	14.2 (7.1-20.5)	9.2 (5.6-23.4)	n.s.	18.2 (11.7-28.7)	7.5 (6.3-19.8)	n.s.	12.0 (6.2-17.8)	11.0 (6.3-26.5)	n.s.
NK cells [x10^6/L]*	**38.7 (17.4-67.2)**	**93.4 (31.4-162.7)**	**0.0001**	49.1 (20.9-72.2)	100.1 (32.8-266.6)	n.s.	**32.6 (15.1-60.7)**	**104.4 (53.1-146.2)**	**0.001**
NK cells of cells [%]*	**0.65 (0.4-1.38)**	**0.4 (0.25-0.75)**	**0.03**	1.0 (0.43-1.58)	0.7 (0.2-0.8)	n.s.	0.4 (0.3-1.13)	0.4 (0.3-0.53)	n.s.
CD39+ NK cells [x10^6/L]*	**5.47 (2.39-11.0)**	**13.8 (6.99-26.1)**	**<0.0001**	7.06 (5.12-15.0)	13.8 (6.91-19.6)	n.s.	**4.05 (2.05-8.51)**	**17.8 (9.15-29.7)**	**0.0002**
CD39+ of NK cells [%]*	14.8 (7.3-34.6)	19.1 (11.7-27.4)	n.s.	20.9 (9.9-53.6)	15.9 (5.7-26.2)	n.s.	**13.6 (6.2-20.8)**	**19.9 (12.3-25.7)**	**0.01**
B cells [x10^6/L]*	**26.5 (16.6-60.2)**	**63.2 (42.2-144.5)**	**0.001**	49.2 (23.4-128.0)	106.4 (63.1-127.5)	n.s.	**22.2 (15.0-31.2)**	**57.4 (40.5-166.6)**	**0.0008**
B cells of cells [%]*	**0.6 (0.3-1.0)**	**0.4 (0.2-0.6)**	**0.004**	**1.0 (0.7-1.6)**	**0.5 (0.3-0.7)**	**0.01**	0.3 (0.3-0.6)	0.4 (0.2-0.5)	n.s.
CD39+B cells [x10^6/L]*	**23.14 (15.45-57.07)**	**61.3 (39.7-134.2)**	**0.0009**	48.1 (20.2-123.6)	103.6 (61.3-120.3)	n.s.	**18.2 (13.5-27.1)**	**51.3 (38.5-154.5)**	**0.0008**
CD39+ of B cells [%]*	95.4 (91.9-97.6)	95.2 (92.4-97.1)	n.s.	95.8 (93.0-97.6)	96.9 (94.3-97.4)	n.s.	95.3 (90.9-97.2)	93.5 (91.9-95.8)	n.s.

*Data are presented as median value (IQR)

IL=interleukin, IFN-g=interferon-γ, TNF-a=tumor necrosis factor-α, CXCL12/SDF-1a= stromal cell-derived factor-α, VEGF-A=vascular endothelial growth factor-A, SCF=stem cell factor, HGF=hepatocyte growth factor, MNC=mononuclear cell, pDC=plasmacytoid dendritic cells, mDC=myeloid dendritic cell,

NKT cells=natural killer T cells, NK cells=natural killer cells; n.s=not significant (bold-p>0.05; Wilcoxon paired))

Table S9: Baseline characteristics of patients with and without a baseline analysis, and divided into GCSF and SMT treatment groups

Parameter	*All patients in accompanying study (n=79)*	*Patients with baseline parameters analyzed (n=60)*	*Patients without baseline parameters analyzed (n=19)*	*p*
Age [years]*	55.0 (51.5-61.0)	56.0 (52.0-61.0)	54.0 (48.0-61.0)	n.s.
Sex [n], male/female	54/25	40/20	14/5	n.s.
BMI*	29.4 (25.1-33.1)	29.4 (25.1-33.3)	28.7 (25.1-31.1)	n.s.
Ascites [n], y/n	77/2	58/2	19/0	n.s.
Albumin [g/L]*	31.0 (27.6-35.7)	32.0 (27.6-36.6)	30.0 (27.5-32.0)	n.s.
Alpha-fetoprotein [ng/mL]*	2.1 (1.0-3.1)	2.1 (1.3-3.1)	1.9 (0.6-3.1)	n.s.
ALT [µkat/L]*	0.68 (0.37-1.20)	0.74 (0.42-1.25)	0.37 (0.2-0.9)	**0.027**
AP [µkat/L]*	2.2 (1.6-2.8)	2.4 (1.7-2.9)	1.5 (1.0-2.3)	**0.044**
AST [µkat/L]*	1.33 (0.82-1.97)	1.36 (0.98-1.97)	0.91 (0.74-2.14)	n.s.
Basophiles [/µL]*	30.2 (11.0-61.8)	30.9 (11.1-68.7)	26.1 (11.9-50.0)	n.s.
Bilirubin [µmol/L]*	342.5 (86.4-488.3)	375.0 (112.9-523.2)	199.0 (56.8-300)	**0.017**
CRP [mg/L]*	32.8 (16.8-57.2)	32.8 (16.5-60.4)	40.3 (18.0-50.33)	n.s.
Eosinophils[/µL]*	165.0 (90.0-326.0)	165.0 (90.0-382.5)	158.9 (825.0-246.5)	n.s.
Erythrocytes [x10^6/µL]*	2.67 (2.40-3.03)	2.69 (2.45-3.08)	2.50 (2.25-2.97)	n.s.
GGT [µkat/L]*	1.49 (0.59-3.54)	1.95 (0.70-3.65)	0.86 (0.45-2.45)	n.s.
HB [mmol/L]*	5.4 (5.0-6.4)	5.5 (5.0-6.5)	5.2 (4.2-5.9)	n.s.
Hematocrit [L/L]*	0.25 (0.23-0.29)	0.26 (0.24-0.29)	0.23 (0.22-0.29)	n.s.
Creatinine [µmol/L]*	188.8 (104.8-270.0)	191.0 (103.5-262.0)	185.0 (116.5-290.7)	n.s.
Leukocytes [x10^9 L]*	10.03 (6.70-14.75)	10.70 (7.00-15.10)	9.44 (4.20-13.60)	n.s.
Monocytes [x10^9/L]*	0.83 (0.50-1.10)	0.90 (0.53-1.10)	0.63 (0.39-0.99)	n.s.
Neutrophils [x10^9/L]*	7.04 (4.29-11.63)	7.81 (4.72-12.27)	6.40 (2.47-8.49)	n.s.
Potassium [mmol/L]*	3.9 (3.5-4.3)	3.9 (3.6-4.3)	3.9 (3.5-4.4)	n.s.
Procalcitonin [ng/mL]*	0.82 (0.47-1.76)	0.89 (0.54-1.92)	0.68 (0.37-1.50)	n.s.
Sodium [mmol/L]*	135.0 (131.0-138.0)	134.0 (131.0-138.0)	136.5 (130.7-139.0)	n.s.
Thrombocytes [x10^9/L]*	71.0 (45.5-124.5)	72.5 (50.0-123.3)	54.0 (33.8-160.3)	n.s.
Urea [mmol/L]*	18.7 (9.4-22.3)	18.7 (12.6-22.1)	18.2 (8.3-24.0)	n.s.
ACLF grade [n], 1/2/3	35/30/13	22/27/10	13/3/3	**0.039**
MELD score*	24.83 (20.25-28.10)	24.93 (20.68-28.25)	22.63 (18.99-25.42)	n.s.
Child Pugh score [n], A/B/C	0/14/64	0/11/48	0/3/16	n.s.
CLIF-C OF*	10 (9-12)	11 (10-12)	10 (8.5-11)	n.s.
HE [n], y/n	59/20	45/15	14/5	n.s.
Death/Transplant within 30d [n]	29/8	22/7	7/1	
Death/Transplant within 90d [n]	40/9	30/8	10/1	
Death/Transplant within 360d [n]	46/13	35/9	11/4	
G-CSF group				
Parameter	**G-CSF (n=40)**	**Patients with baseline parameters analyzed G-CSF group (n=34)**	**Patients without baseline parameters analyzed G-CSF group(n=6)**	**p**
Age [years]*	54.5 (51.0-59.0)	55.0 (52.0-58.8)	48.0 (42.5-60.0)	n.s.

Sex [n], male/female	24/16	22/12	2/4	n.s.
BMI*	29.4 (26.2-31.2)	29.4 (26.2-31.2)	30.7 (29.2-31.2)	n.s.
Ascites [n], y/n	39/1	33/1	6/0	n.s.
Albumin [g/L]*	30.9 (27.3-35.8)	30.9 (27.6-36.0)	28.5 (26.1-30.8)	n.s.
Alpha-fetoprotein [ng/mL]*	2.1 (1.4-2.9)	2.1 (1.7-3.0)	1.4 (1.0-2.3)	n.s.
ALT [µkat/L]*	0.68 (0.37-1.03)	0.69 (0.42-1.12)	0.26 (0.18-0.69)	n.s.
AP [µkat/L]*	2.2 (1.8-2.6)	2.2 (1.8-2.6)	2.1 (1.9-2.3)	n.s.
AST [µkat/L]*	1.33 (0.93-1.84)	1.31 (0.98-1.93)	1.60 (0.96-1.73)	n.s.
Basophiles [/µL]*	31.6 (12.5-62.7)	31.3 (13.7-67.0)	37.6 (8.6-47.8)	n.s.
Bilirubin [µmol/L]*	365.2 (93-486.8)	373.9 (152.8-500.8)	129.8 (54.9-324.3)	n.s.
CRP [mg/L]*	31.7 (17.2-59.4)	32.3 (12.0-62.0)	30.9 (21.1-50.5)	n.s.
Eosinophils[/µL]*	161.3 (90.0-284.0)	161.3 (90.0-319.7)	161.5 (30.8-239.0)	n.s.
Erythrocytes [x10^6/µL]*	2.67 (2.45-2.96)	2.63 (0.56-3.03)	2.91 (24.5-29.8)	n.s.
GGT [µkat/L]*	1.42 (0.62-3.44)	1.59 (0.67-3.09)	1.34 (0.76-3.22)	n.s.
HB [mmol/L]*	5.7 (5.0-6.2)	5.4 (5.0-6.3)	5.5 (4.8-6.0)	n.s.
Hematocrit [L/L]*	0.25 (0.24-0.29)	0.25 (0.24-0.29)	0.25 (0.23-0.29)	n.s.
Creatinine [µmol/L]*	185.8 (89.7-242.8)	191.8 (89.8-246.3)	153.0 (97.9-202.3)	n.s.
Leukocytes [x10^9 L]*	10.90 (7.35-15.02)	10.70 (7.38-15.08)	12.45 (6.03-14.45)	n.s.
Monocytes [x10^9/L]*	0.90 (0.57-1.08)	0.91 (0.62-1.05)	0.69 (0.45-1.02)	n.s.
Neutrophils [x10^9/L]*	7.75 (4.95-11.67)	7.81 (5.05-10.91)	6.45 (2.91-11.92)	n.s.
Potassium [mmol/L]*	3.9 (3.5-4.2)	3.9 (3.6-4.2)	3.5 (3.4-4.2)	n.s.
Procalcitonin [ng/mL]*	0.68 (0.46-1.45)	0.78 (0.45-2.11)	0.6 (0.5-0.7)	n.s.
Sodium [mmol/L]*	134.6 (130.8-137.3)	134.6-131.3-137.8)	133.2 (130.3-135.8)	n.s.
Thrombocytes [x10^9/L]*	76.0 (50.0-149.5)	81.0 (50-13.8)	70.0 (52.0-21.3)	n.s.
Urea [mmol/L]*	16.2 (8.5-21.0)	17.2 (8.5-21.4)	12.4 (7.8-19.3)	n.s.
ACLF grade [n], 1/2/3	17/18/5	12/17/5	5/1/0	n.s.
MELD score*	24.9 (21.9-27.7)	24.9 (22.9-27.9)	19.1 (18.9-21.7)	n.s.
Child Pugh score [n], A/B/C	0/9/31	0/8/26	0/1/5	n.s.
CLIF-C OF*	10 (9-11)	10.5 (10.0-12.0)	8.0 (8.0-9.5)	**0.045**
HE [n], y/n	29/11	25/9	4/2	n.s.
Death/Transplant within 30d [n]	14/3	12/3	2/0	
Death/Transplant within 90d [n]	16/5	15/4	1/1	
Death/Transplant within 360d [n]	21/5	18/4	3/1	
SMT group				
Parameter	**SMT (n= 39)**	**Patients with baseline parameters analyzed SMT group (n=26)**	**Patients without baseline parameters analyzed SMT group(n=13)**	**p**
Age [years]*	56.0 (52.0-63.5)	56.5 (52.3-64.0)	55.0 (52.0-59.0)	n.s.
Sex [n], male/female	30/9	18/8	12/1	n.s.
BMI*	28.7 (24.7-33.5)	30.0 (22.5-34.0)	27.1 (25.0-30.4)	n.s.
Ascites [n], y/n	38/1	25/1	13/0	n.s.
Albumin [g/L]*	32.0 (27.7-34.2)	32.8 (27.2-37.2)	31.0 (29.0-33.1)	n.s.
Alpha-fetoprotein [ng/mL]*	2.1 (1.0-3.7)	2.1 (1.0-5.6)	2.0 (0.4-3.4)	n.s.
ALT [µkat/L]*	0.70 (0.38-1.25)	0.79 (0.48-1.25)	0.40 (0.27-1.10)	n.s.
AP [µkat/L]*	2.2 (1.3-2.9)	2.5 (1.7-3.4)	1.2 (1.0-2.2)	**0.044**
AST [µkat/L]*	1.28 (0.79-2.39)	1.54 (0.98-2.10)	0.88 (0.72-2.53)	n.s.
Basophiles [/µL]*	30.0 (10.4-60.0)	30.4 (10.0-73.0)	21.1 (14.8-51.6)	n.s.

Bilirubin [µmol/L]*	316.8(81.6-502.2)	434.4 (86.3-536.2)	218.8 (80.0-270.0)	n.s.
CRP [mg/L]*	38.3 (17.1-49.3)	37.5 (21.7-45.1)	42.2 (16.4-49.8)	n.s.
Eosinophils[/µL]*	172.0 (90.0-377.4)	197.6 (90.0-428.8)	158.9 (87.5-239.0)	n.s.
Erythrocytes [x10⁶/µL]*	2.70 (2.40-3.26)	2.83 (2.58-3.27)	2.41 (2.10-2.80)	n.s.
GGT [µkat/L]*	1.71 (0.55-3.43)	2.33 (1.08-3.81)	0.74 (0.36-1.84)	**0.032**
HB [mmol/L]*	5.3 (4.8-6.6)	5.6 (5.1-6.6)	5.1 (4.2-5.5)	n.s.
Hematocrit [L/L]*	0.26 (0.23-0.29)	0.26 (0.23-0.29)	0.23 (0.21-0.29)	n.s.
Creatinine [µmol/L]*	195 (115.3-279.8)	191.0 (113.0-263.0)	212.0 (119.0-329.0)	n.s.
Leukocytes [x10⁹ L]*	8.70 (5.63-14.25)	8.80 (6.00-15.00)	8.30 (4.30-10.25)	n.s.
Monocytes [x10⁹/L]*	0.77 (0.39-1.10)	0.82(0.45-1.11)	0.63 (0.36-0.98)	n.s.
Neutrophils [x10⁹/L]*	6.64 (4.14-11.52)	8.21 (4.60-12.53)	6.39 (2.08-7.68)	n.s.
Potassium [mmol/L]*	3.92 (3.59-4.39)	3.76 (3.60-4.37)	3.95 (3.58-4.60)	n.s.
Procalcitonin [ng/mL]*	1.12 (0.56-1.78)	1.12 (0.61-1.67)	0.97 (0.22-2.25)	n.s.
Sodium [mmol/L]*	135 (131.3-138.8)	133.1 (131.0-138.0)	137.0(133.0-142.0)	n.s.
Thrombocytes [x10⁹/L]*	63.0 (32.0-102.0)	68.0 (42.8-99.0)	54.0 (30.0-113.0)	n.s.
Urea [mmol/L]*	19.5 (14.8-26.7)	19.6 (15.6-24.5)	19.0 (9.8-26.7)	n.s.
ACLF grade [n], 1/2/3	18/12/8	10/10/5	8/2/3	n.s.
MELD score*	24.6 (20.1-29.4)	25.2 (20.2-29.6)	24.4 (20.1-25.4)	n.s.
Child Pugh score [n], A/B/C	0/5/33	0/3/22	0/2/11	n.s.
CLIF-C OF*	11 (9-12)	11.0 (10.0-12.8)	11.0 (9.0-11.0)	n.s.
HE [n], y/n	30/9	20/6	10/3	n.s,
Death/Transplant within 30d [n]	15/5	10/4	5/1	
Death/Transplant within 90d [n]	23/5	15/4	8/1	
Death/Transplant within 360d [n]	25/8	17/5	8/3	

*Data are presented as median value (IQR)

ALT=alanine aminotransferase, AP=alkaline phosphatase, AST=aspartate transaminase, CRP=C-reactive protein, GGT=γ-gutamyltransferase, HB=haemoglobin, CLIF-C OF score = CLIF-C organ failure score,; n.s=not significant (p>0.05; Mann-Whitney or Fischer)

3. Supplementary figure legends

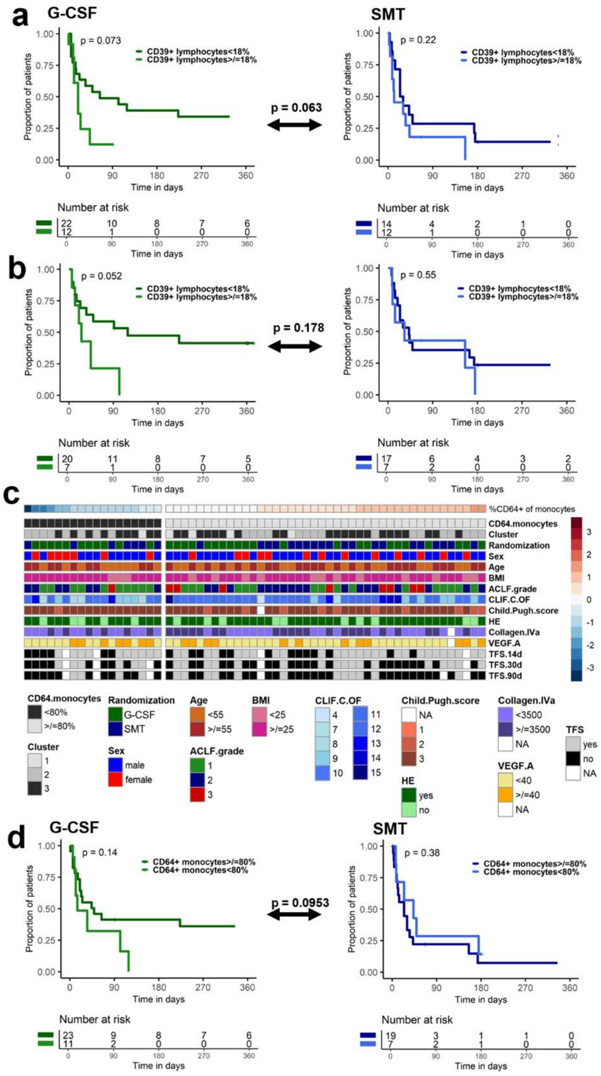

Supplementary Fig. 1: Survival curves of patients classified by relative levels of CD39+ lymphocytes and CD64+ monocytes. Survival analysis of patients classified by relative levels of CD39+ lymphocytes at baseline (a) and V2 (b) P values of cell population treatment interaction was p=0.063 and 0.178. Heatmap (c) and survival analysis (d) of patients classified by relative levels of CD64+ monocytes. P value of cell population treatment interaction was p=0.095. The statistical significances of the survival curves were analyzed using Kaplan-Meier method and Log-rank test.

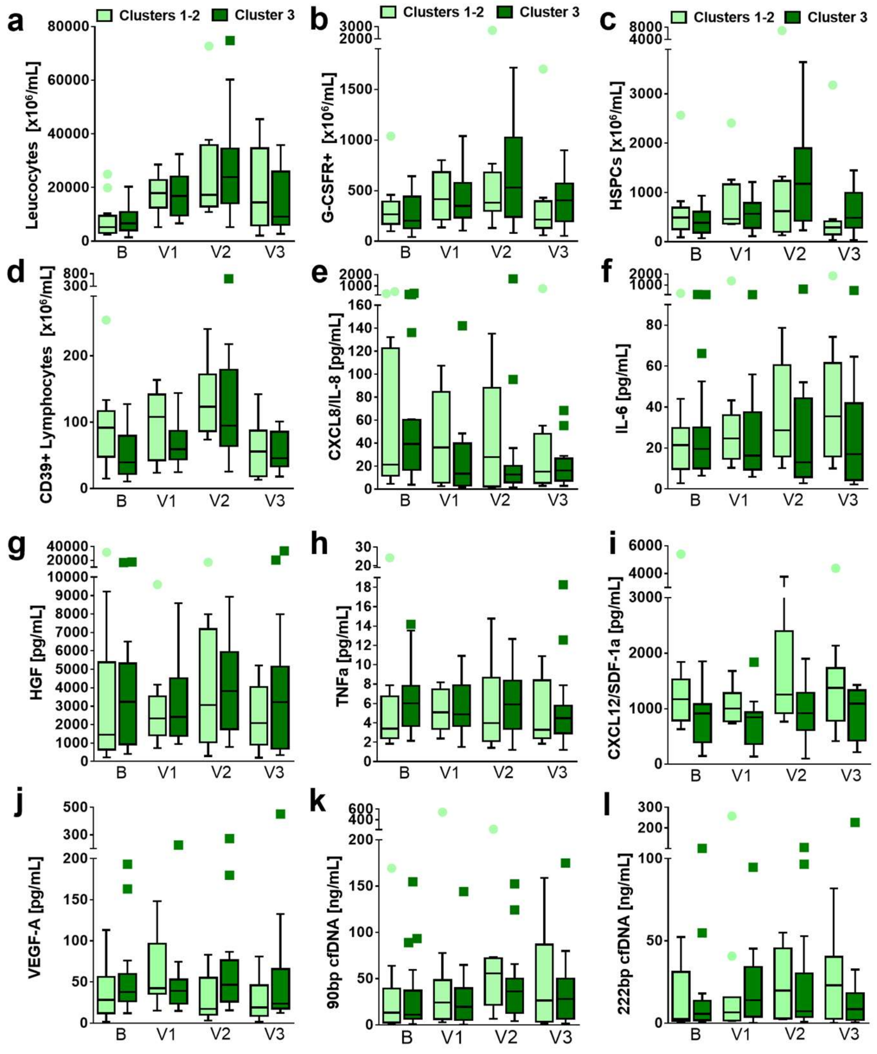

Supplementary Fig. 2: Progression of cell populations and cytokines in G-CSF treated patients. Progression of (a) Leucocytes, (b) G-CSFR+ cells, (c) HSPCs, (d) CD39+ lymphocytes, (e) CXCL8/IL-8, (f) IL-6, (g) HGF, (h) TNFa, (i) CXCL12/SDF-1a (j) VEGF-A, (k) 90bp cfDNA and (l) 222bp cfDNA in clusters 1-2 (light green) cluster 3 (dark green) in G-CSF treated patients.

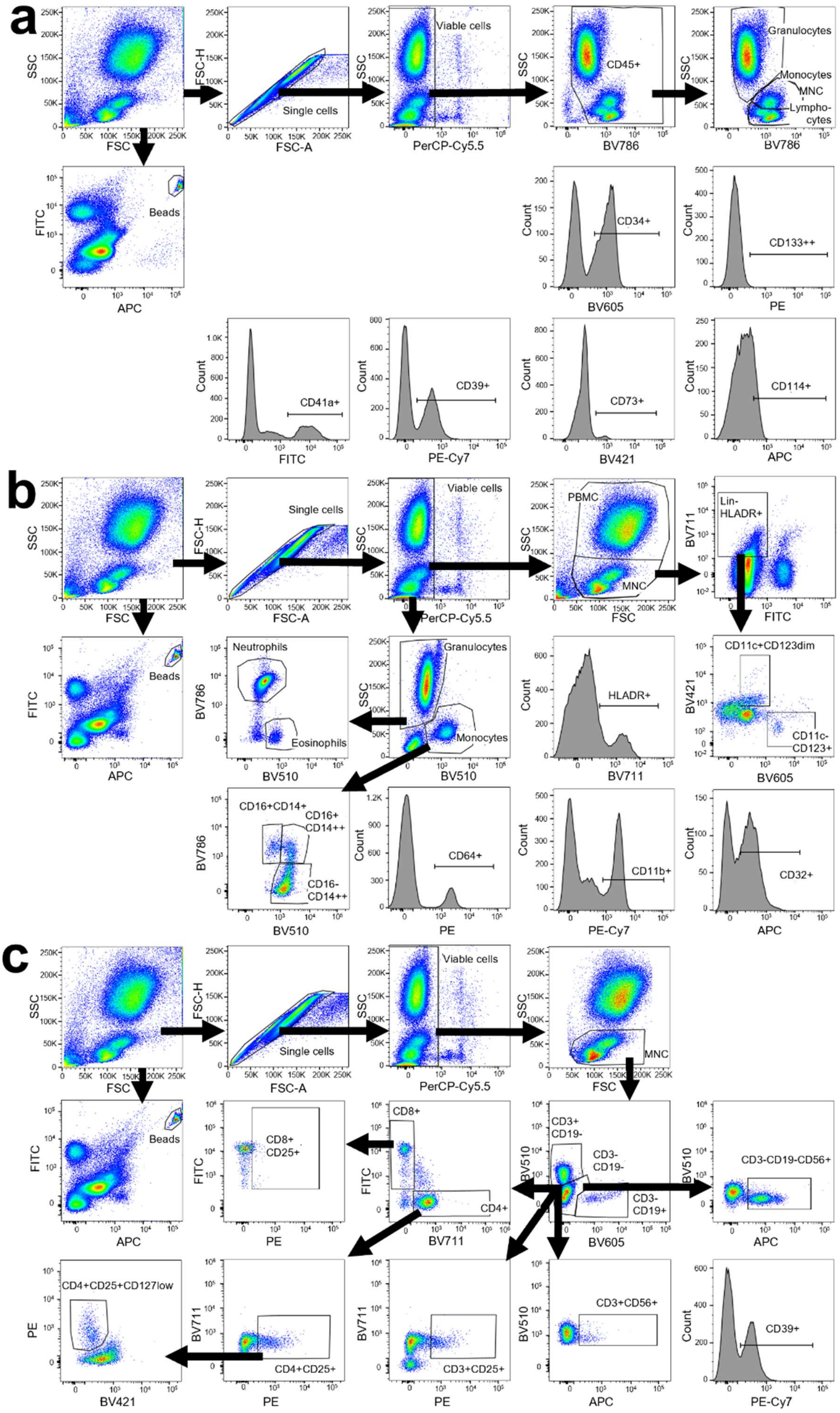
a
b
c
SSC
FSC
FSC-H
FSC-A
Single cells
PerCP-Cy5.5
Viable cells
BV786
CD45+
Granulocytes
Monocytes
MNC
Lympho-
cytes
FITC
APC
Beads
Count
BV605
CD34+
PE
CD133++
CD41a+
PE-Cy7
CD39+
BV421
CD73+
CD114+
PBMC
BV711
Lin-
HLADR+
BV510
Neutrophils
Eosinophils
HLADR+
CD11c+CD123dim
CD11c-
CD123+
CD16+CD14+
CD16+
CD14++
CD16-
CD14++
CD64+
CD11b+
CD32+
CD8+
CD25+
CD4+
CD3+
CD19-
CD3-
CD19-
CD3-
CD19+
CD3-CD19-CD56+
CD4+CD25+CD127low
CD4+CD25+
CD3+CD25+
CD3+CD56+

Supplementary Fig. 3: Gating strategies for the flow cytometry analysis of patient blood cells. Representative examples of cell populations for the (a) HSPCs panel, (b) monocytes panel, and (c) lymphocyte panel. The markers in the grey histograms were applied to all gated populations in each panel.

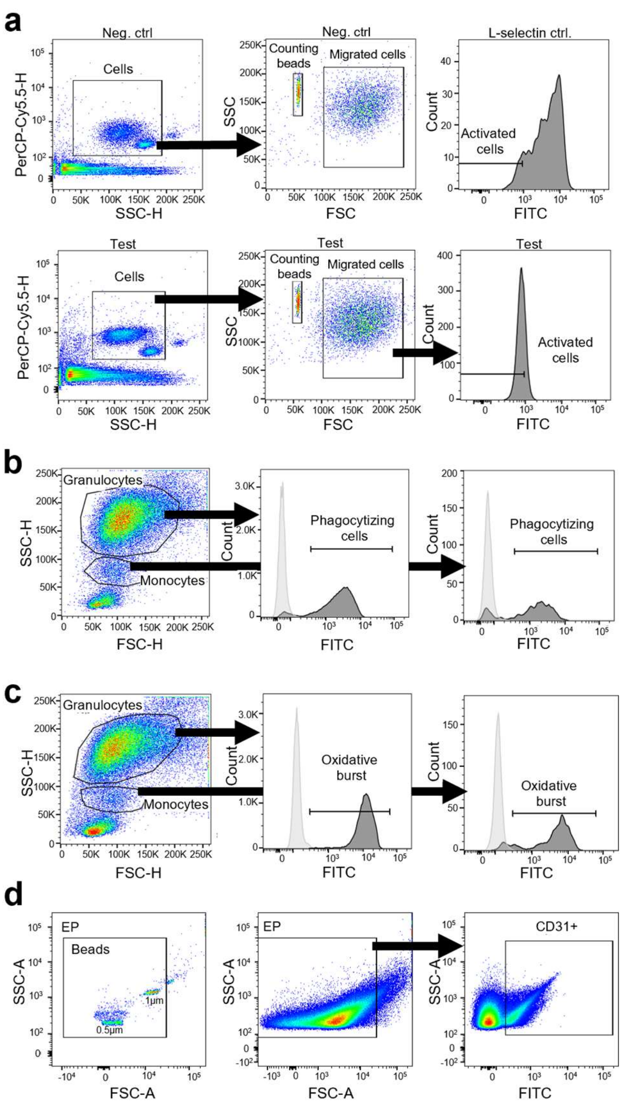

Supplementary Fig. 4: Gating strategies for the functional assays and EPs. Representative examples for the flow cytometry analysis of Migratest (a), Phagotest (b), and Phagoburst (c) assay. Representative examples for the flow cytometry analysis of CD31+ EPs(d).

1. Supplementary references

1. Engelmann C, Herber A, Franke A, Bruns T, Reuken P, Schiefke I, et al. Granulocyte-colony stimulating factor (G-CSF) to treat acute-on-chronic liver failure: A multicenter randomized trial (GRAFT study). Journal of Hepatology 2021.
2. Moreau R, Jalan R, Gines P, Pavesi M, Angeli P, Cordoba J, Durand F, et al. Acute-on-chronic liver failure is a distinct syndrome that develops in patients with acute decompensation of cirrhosis. Gastroenterology 2013;144:1426-1437, 1437 e1421-1429.
3. Jalan R, Saliba F, Pavesi M, Amoros A, Moreau R, Gines P, Levesque E, et al. Development and validation of a prognostic score to predict mortality in patients with acute-on-chronic liver failure. J Hepatol 2014;61:1038-1047.
4. Karlas T, Weise L, Kuhn S, Krenzien F, Mehdorn M, Petroff D, et al. Correlation of cell-free DNA plasma concentration with severity of non-alcoholic fatty liver disease. Journal of Translational Medicine 2017;15:106.
5. Krenzien F, Katou S, Papa A, Sinn B, Benzing C, Feldbrügge L, et al. Increased Cell-Free DNA Plasma Concentration Following Liver Transplantation Is Linked to Portal Hepatitis and Inferior Survival. Journal of Clinical Medicine 2020;9.
6. Breitbach S, Tug S, Helmig S., Zahn, D., Kubiak, T., Michal, M., et al. Direct quantification of cell-free, circulating DNA from unpurified plasma. PLoS One. 2014;9(3):e87838.
7. Kamali K, Schmelzle M, Kamali C, Brunnbauer P, Splith K, Leder A, et al. Sensing Acute Cellular Rejection in Liver Transplant Patients Using Liver-Derived Extracellular Particles: A Prospective, Observational Study. Frontiers in Immunology 2021;12.
8. Kornek M, Popov Y, Libermann TA, Afdhal NH, Schuppan D. Human T cell microparticles circulate in blood of hepatitis patients and induce fibrolytic activation of hepatic stellate cells. Hepatology 2011;53:230–42.
9. Antoniades CG, Berry PA, Davies ET, et al. Reduced monocyte HLA-DR expression: a novel biomarker of disease severity and outcome in acetaminophen-induced acute liver failure. Hepatology 2006;44:34-43.
10. Yang J, Yi P, Wei L, et al. Phenotypes and clinical significance of circulating CD4(+)CD25(+) regulatory T cells (Tregs) in patients with acute-on-chronic liver failure (ACLF). J Transl Med 2012;10.
11. Deaglio S, Dwyer KM, Gao W, et al. Adenosine generation catalyzed by CD39 and CD73 expressed on regulatory T cells mediates immune suppression. J Exp Med 2007;204:1257-1265.

12. Jilma B, Hergovich N, Homoncik M, et al. Granulocyte colony-stimulating factor (G-CSF) downregulates its receptor (CD114) on neutrophils and induces gelatinase B release in humans. Br J Haematol 2000;111:314-320.
13. Garg V, Garg H, Khan A, et al. Granulocyte Colony–Stimulating Factor Mobilizes CD34+ Cells and Improves Survival of Patients With Acute-on-Chronic Liver Failure. Gastroenterology 2012;142:505-512.e1.
14. Melve GK, Ersvaer E, Eide GE, Kristoffersen EK, Bruserud Ø. Peripheral Blood Stem Cell Mobilization in Healthy Donors by Granulocyte Colony-Stimulating Factor Causes Preferential Mobilization of Lymphocyte Subsets. Front Immunol. 2018 May 2;9:845. doi: 10.3389/fimmu.2018.00845. PMID: 29770133; PMCID: PMC5941969.
15. Khanam A, Trehanpati N, Garg V, Kumar C, Garg H, Sharma BC, Sarin SK. Altered frequencies of dendritic cells and IFN-gamma-secreting T cells with granulocyte colony-stimulating factor (G-CSF) therapy in acute-on- chronic liver failure. Liver Int. 2014 Apr;34(4):505-13. doi: 10.1111/liv.12415. PMID: 24754047.
16. Szabo G. Pathogenesis of Acute-on-Chronic Liver Failure in Patients With Infection. Clin Liver Dis (Hoboken). 2019 Oct 9;14(3):103-106. doi: 10.1002/cld.826. PMID: 31632659; PMCID: PMC6784801.
